# Distinct biophysical and chemical mechanisms governing sucrose mineralization and soil organic carbon priming in biochar amended soils: evidence from 10 years of field studies

**DOI:** 10.1007/s42773-024-00327-0

**Published:** 2024-05-22

**Authors:** Haoli Zhang, Tao Ma, Lili Wang, Xiuling Yu, Xiaorong Zhao, Weida Gao, Lukas Van Zwieten, Bhupinder Pal Singh, Guitong Li, Qimei Lin, David R. Chadwick, Shenggao Lu, Jianming Xu, Yu Luo, David L. Jones, Peduruhewa H. Jeewani

**Affiliations:** 1 College of Land Science and Technology, China Agriculture University, Yuanmingyuan West Road, Beijing, 100193 China; 2 Crop Research Institute, Guangxi Agricultural Vocational University, Guangxi, China; 3 Agro-Environmental Protection Institute, Ministry of Agriculture and Rural Affairs, Tianjin, 300191 China; 4 Institute of Soil and Water Resources and Environmental Science, Zhejiang Provincial Key Laboratory of Agricultural Resources and Environment, Zhejiang University, Hangzhou, 310058 China; 5 School of Environmental and Natural Sciences, Environment Centre Wales, Bangor University, Gwynedd, LL57 2UW UK; 6 NSW Department of Primary Industries, Wollongbar Primary Industries Institute, Wollongbar, NSW 2477 Australia; 7 Soils West, Centre for Sustainable Farming Systems, Food Futures Institute, Murdoch University, 90 South Street, Murdoch, WA 6150 Australia

**Keywords:** X-ray CT, Porosity, Aggregates, Microbial community, Decadal-scale field study

## Abstract

While many studies have examined the role of biochar in carbon (C) accrual in short-term scale, few have explored the decadal scale influences of biochar on non-biochar C, e.g., native soil organic C (SOC) and added substrate. To address this knowledge gap, soils were collected from decade-old biochar field trials located in the United Kingdom (Cambisol) and China (Fluvisol), with each site having had three application rates (25–30, 50–60 and 75–100 Mg ha^−1^) of biochar plus an unamended Control, applied once in 2009. We assessed physicochemical and microbial properties associated with sucrose (representing the rhizodeposits) mineralization and the priming effect (PE) on native SOC. Here, we showed both soils amended with biochar at the middle application rate (50 Mg ha^−1^ biochar in Cambisol and 60 Mg ha^−1^ biochar in Fluvisol) resulted in greater substrate mineralization. The enhanced accessibility and availability of sucrose to microorganisms, particularly fast-growing bacterial genera like *Arenimonas, Spingomonas,* and *Paenibacillus* (r-strategists belonging to the Proteobacteria and Firmicutes phyla, respectively), can be attributed to the improved physicochemical properties of the soil, including pH, porosity, and pore connectivity, as revealed by synchrotron-based micro-CT. Random forest analysis also confirmed the contribution of the microbial diversity and physical properties such as porosity on sucrose mineralization. Biochar at the middle application rate, however, resulted in the lowest PE (0.3 and 0.4 mg of CO_2_-C g soil^−1^ in Cambisol and Fluvisol, respectively) after 53 days of incubation. This result might be associated with the fact that the biochar promoted large aggregates formation, which enclosed native SOC in soil macro-aggregates (2–0.25 mm). Our study revealed a diverging pattern between substrate mineralization and SOC priming linked to the biochar application rate. This suggests distinct mechanisms, biophysical and physicochemical, driving the mineralization of non-biochar carbon in a field where biochar was applied a decade before.

**Supplementary Information:**

The online version contains supplementary material available at 10.1007/s42773-024-00327-0.

## Highlights

- The application of biochar at a moderate rate (50–60 Mg ha^−1^) resulted in increased labile C mineralization and decreased priming of native SOC.
- Biochar amendments led to the reconfiguration of physicochemical properties, including pH, porosity, and the formation of aggregates.
- Modified microbial community structure towards more r-strategists caused greater sucrose mineralization.
- Biochar incorporation rate governed the C dynamics via (biophysical vs physicochemical mechanisms) in long-term biochar amended fields.

**Graphical Abstract:**

**Supplementary Information:**

The online version contains supplementary material available at 10.1007/s42773-024-00327-0.

## Graphical abstract

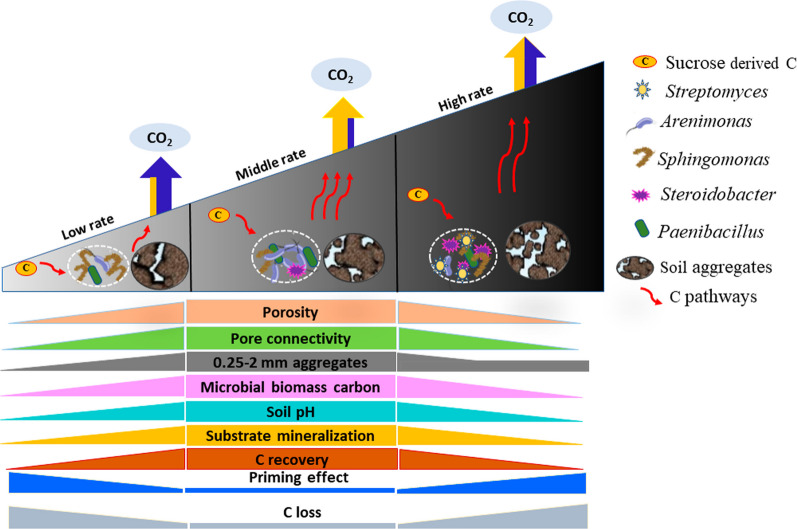

## Introduction

Sequestration of soil organic carbon (SOC) is one mechanism that can offset greenhouse gas emissions, while improving the physicochemical and biological properties of soil (Lehmann et al. 2015). Biochar was a stable additive for long-term soil carbon (C) storage (Lehmann et al. 2011). The amendment of soil with biochar has been widely proposed for increasing SOC due to its resistance to biotic and abiotic degradation. Biochar can increase and/or decrease the turnover rates of both existing SOC and low molecular weight C compounds (e.g., rhizodeposits); therefore, biochar may facilitate the divergent effects on mineralization of SOC (Whitman et al. 2014; Keith et al. 2015). However, the C sequestration potential of biochar amended soils and its interaction with native SOC and low molecular weight C compounds may change as biochar ages in soil, and related information is lacking.

There are, however, interactions between biochar and non-biochar carbon (NBC), e.g., SOC, rhizodeposits and crop residues (Luo et al. 2011; Weng et al. 2017). For example, biochar can stimulate a short-term positive priming effect (PE), with up to five-fold losses of native SOC over a period of 87 days (Luo et al. 2011; Maestrini et al. 2015; Wang et al. 2016), or induce a negative PE (Li et al. 2018; Yu et al. 2020) whereby SOC is further stabilized. The literature tends to agree that the positive priming effect is short-term, and that longer-term negative priming effects stabilize SOC (Weng et al. 2015, 2022). Similarly, when labile sources of C (e.g., glucose) are added into biochar amended soils, both positive and negative PE effects on SOC mineralization were widely reported (Zhang et al. 2019; Zimmerman and Ouyang 2019). These inconsistent results of biochar related C mineralization can be attributed to the differences in the intrinsic properties of substrate (e.g., hydrophobicity), biochar itself, as well as the changes in edaphic properties of soil (Luo et al. 2017; Zheng et al. 2021). The legacy effects on the soil changes after the application of biochar that affect the dynamics of non-biochar C determine its overall sequestration effects of biochar, and it is therefore important to have a comprehensive understanding of biotic and abiotic mechanisms underpinning the fate of non-biochar C, i.e., new input of C or native SOC in biochar amended soils.

Biochar has been shown to provide improvements in soil physical properties including bulk density, aggregation, intra-aggregate pores and pore connectivity (Zong et al. 2015; Yu et al. 2018; Yu and Lu 2019), due to its low density and high specific surface area (Laird et al. 2010; Singh and Cowie 2014; Yu et al. 2018). Biochar has also been observed to increase soil aggregation by 16% (Islam et al. 2021). It is generally understood that soil aggregates confine plant debris in the core of the microaggregates, thus stabilizing new C and protecting it from microbial mineralization, whereas existing SOC is more commonly protected in occluded forms within microaggregates (Blanco-Canqui and Lal 2004).

The modifications of physicochemical properties caused by biochar can facilitate the growth of microbes and alter soil microbial communities (Nielsen et al. 2014). Present short-term studies both in the laboratory and in the field have showed that after incorporation into soil, biochar could induce significant changes in soil microbial biomass, activity and community composition (Farrell et al. 2013; Luo et al. 2013; Ameloot et al. 2014; Gomez et al. 2014). Application of biochar can facilitate with soil microbial assembly through providing high C inputs and improving resource accessibility for microbes due to porous structure (Lehmann and Joseph 2009; Smith et al. 2010). Biochar can also provide suitable habitats for microbial survival and protection from predators due to compartmentalisation (Pietikäinen et al. 2000; Lehmann et al. 2011; Luo et al. 2013). In contrast, biochar may also inhibit microbial activity via adsorbing/stabilizing organic substances due to its large surface area or aggregate formation (Kasozi et al. 2010; Rutigliano et al. 2014). A modification of the physiochemical properties of microbial ecological niche (e.g. soil pH, C and nutrient availability) can have negatively effect on certain microbial communities (Hardy et al. 2019). For instance, two years after biochar incorporation, Ameloot et al. (2014) observed a lowered soil microbial activity, abundance and shifted community composition in biochar amended soil with a rate of  49 Mg ha^−1^ compared to the non-amended soil. Biochar can sequester C by shifting the bacterial community towards low C turnover bacterial taxa (e.g., Acidobacteria and delta Proteobacteria), which may also indicate a microbiological mechanism of stabilization of SOC under long-term biochar addition (Chen et al. 2019; Liao et al. 2019). Whether the changes in soil properties and microbial communities caused by biochar application persist for years afterwards is unclear, as most field studies haven't investigated beyond a few years.

Despite an increasing number of studies that show biochar effects on soil physicochemical and microbial properties, a paucity of information still exists on the impact of these soil abiotic and biotic changes on C dynamics (exogenous C inputs or SOC). This is especially the case for longer-term field-based studies. To address this knowledge gap, we utilized soil from decade-long biochar field trials in the United Kingdom (Cambisol) and China (Fluvisol), where three biochar application rates were applied. The objectives of this study were to determine the effects of biochar, with a decadal scale, on (i) soil physicochemical properties (e.g. pore traits as analyzed by synchrotron-based X-ray CT), (ii) microbial community structure (analyzed by sequencing), and (iii) how these biochar-induced abiotic and biotic changes affect C mineralization (exogenous sucrose mineralization and sucrose-induced SOC priming). We hypothesized that: (i) the presence of biochar would increase sucrose mineralization due to higher accessibility to microorganisms, particularly r-strategists, via enhanced soil porosity and pore connectivity, and (ii) biochar would lower substrate-induced SOC priming, resulting from changes in soil aggregation that protect SOC from microbial mineralization. This study aims to improve our understanding of the mechanisms underpinning C mineralization and sequestration potential in biochar-amended soils by quantifying native SOC, substrate derived C mineralization patterns and changes in soil physicochemical properties, microbial communities, and their interactions.

## Materials and methods

### Site description

The soils were collected from two long-term (10 years) biochar field trials: (i) the Long-Term Biochar Agronomic (LTBA) field trial at Bangor University in Abergwyngregyn, Wales, United Kingdom (53° 23′ 37″ N, 4° 01′ 44″ E), and (ii) the trial at the Shang-Zhuang experimental station of the China Agricultural University in Haidian district, Beijing, China (40° 08′ 21″ N, 116° 10′ 52″ E). The soil at the UK site was a sandy clay loam and classified as a Eutric Cambisol (IUSS Working Group WRB, 2015). The mean annual rainfall is 1250 mm, and the mean annual air temperature is 10.6 °C. Here**,** mixed-hardwood biochar pyrolyzed at 450 °C for 48 h was added to the soil surface and power harrowed into the soil in 2009 at the application rates of 0, 25, 50, and 75 Mg ha^−1^ (Additional file 1: Table S6). The soil collected from China was classified as a Eutric Fluvisol (IUSS Working Group WRB, 2015) with the application of biochar at 0, 30, 60, and 90 Mg ha^−1^ in 2009. It has a typical continental monsoon climate, with 400 mm annual precipitation and 11.6 °C annual air temperature. Biochar was produced with woodchip waste from mushroom production and was pyrolyzed at 400 °C for 48 h (Additional file 1: Table S7).

### Experimental setup

An incubation experiment was conducted over 53 days to investigate the priming effects of exogenous substrate (sucrose) application to soil. The treatments of the experiment were control (0 Mg ha^−1^), low rate (25–30 Mg ha^−1^), middle rate (50–60 Mg ha^−1^) and high rate (75–90 Mg ha^−1^) for both Cambisol and Fluvisol, respectively, with three replicates (n = 3). Soil cores (~ 500 g, δ^13^C = −26.36 ~ −0.01‰ and δ^13^C = −24.78 ~ −0.07‰ Cambisol and Fluvisol respectively) were collected from the 0–10 cm layer of each replicated plot at both sites. The soil cores were packed in plastic bags to preserve them in optimal conditions, then transported to Zhejiang University, China. The water holding capacity (WHC) of soil cores were adjusted without disturbing the structures and preincubated at 25 °C for 7 days before starting the experiments, to avoid any early sampling effects on soil processes (Kemmitt et al. 2008). Sucrose, a commonly used, easily available, low molecular weight carbon compound representing rhizomatous deposition, was used as a substrate and was added at a rate of 10 mg sucrose g^−1^ soil to represent a realistic annual residual C input to soil (Nottingham et al. 2009). After the preincubation, 1% sucrose solution (δ^13^C = −11.97 ± 0.12‰) was applied into soil column using a pipette (the content of added water was accounted to reach 40% WHC). The un-amended soil cores were treated in the same way with distilled water without sucrose. Then soil cores were incubated inside a 1000 mL glass jar containing 10 mL water in the base to maintain humidity**.** For the collection of CO_2_, a 25 mL glass vial containing 20 mL of 1 M NaOH was placed in each large jar. The jars were then sealed with rubber bungs and incubated in a randomized block design at 25 ℃ for 53 days. Three additional blanks, consisting of jars with only water and NaOH, were included for quality control purposes. The NaOH vials were replaced after 1, 3, 7, 14, 28 and 53 days. The incubation jars were opened periodically to maintain aerobic conditions (Jeewani et al. 2021).

### Soil properties

The initial soil physiochemical and biological properties were measured. Soil pH was measured using a soil:water ratio of 1:2.5 (w/w) (Ling et al. 2022). Total soil C (TC) and N (TN) concentrations were determined by dry combustion (Perkin Elmer EA 2400, Shelton, CT, USA) (Jeewani et al. 2021). Microbial biomass carbon (MBC) was determined by fumigation extraction (Vance et al. 1987; Wu et al. 1990). The K_2_SO_4_ extractable organic C was quantified using an organic carbon auto-analyzer (Shimadzu, Analytical Sciences, Kyoto, Japan). The cation exchange capacity (CEC) was determined by a modified NH_4_^+^-acetate compulsive displacement method (Gaskin et al. 2008). Soil bulk density (BD) was measured for both sites using a core sampling method (Casanova et al. 2016). Dry sieving method was used for soil fractionation which was adapted from Elliott (1986). At the end of the incubation, 20 g representative samples were passed through a series of three sieve sizes (0.053 mm, 0.25 mm, and 2 mm,) to isolate four aggregate size fractions: (i) > 2 mm (large macroaggregates); (ii) 0.25–2 mm (small macroaggregates); (iii) 0.053–0.25 mm (microaggregates); (iv) < 0.053 mm (silt and clay fraction). Meanwhile, stones, roots and other impurities were eliminated in the soil samples. Then the whole series of sieves were moved up and down for 7 min at 30 cycle min^−1^. The aggregate fractions retained on each sieve were weighed. The natural ^13^C abundance in the soils, biochar and soil aggregate fractions were determined using an isotope ratio mass spectrometer (DELTA V plus IRMS) coupled with an elemental analyzer (EA NA1500—EA 1110 device, Carlo Erba and Thermo Fisher Scientific Bremen, Germany).

### Analysis of CO_2_-C

An aliquot (5 ml) from the collected NaOH trap was mixed with 10 ml water and titrated against 0.5 M HCl using an Easy Plus auto titrator (Mettler Toledo, Greifensee, Switzerland). The ^13^C-CO_2_ was analysed according to methods of Aoyama et al. (2000). In brief, an 8 mL aliquot from the NaOH trap was added to 8 mL 1.5 M BaCl_2_ in a 50 ml centrifuge tube, and  the solution was incubated at 25 °C for 0.5 h before being centrifuged at 4000 × *g* for 10 min. The resulting BaCO_3_ precipitate was carefully rinsed with water three times, placed in a refrigerator for 24 h and freeze-dried overnight. The precipitates were collected, and ca. 0.200 mg was accurately weighed into tin boats (4*4*11 mm). The prepared samples were then analyzed for δ^13^C using an Elementar vario micro cube elemental analyzer coupled with GV isoprime 100 isotope ratio mass spectrometer (GV Instruments, United Kingdom).

### δ^13^C calculations

The mineralization of sucrose (C4-derived) was distinguished from biochar amended soil (C3 derived) mineralization based on the changes in the stable isotopic composition (δ^13^C) over time. The standard equation for determining δ^13^C is derived from:

1 $${\updelta }^{13}{\text{C}}\left(\permil\right)=[\left(\frac{{\text{Rsample}}}{{{\text{R}}}_{{\text{VPDB}}}}\right)-1]\times 1000$$

The value of ^13^C and ^12^C atomic ratio of the standard material was 0.0112372, where R_sample_ is the mass ratio of ^13^C to ^12^C of the sample, and R_PDB_ is the mass ratio of ^13^C to ^12^C of the Vienna Peedee belemnite (V_PDB_) standard (Craig 1953).

2 $${{\text{C}}}_{4}={{\text{C}}}_{{\text{t}}}\times \left(\frac{{\delta }_{t}- {\delta }_{3}}{{\delta }_{4}-{\delta }_{3}}\right)$$

3 $${{\text{C}}}_{{\text{t}}}={{\text{C}}}_{3}+{{\text{C}}}_{4}$$

where C_t_ is the total CO_2_, C_3_ and C_4_ are the respective amounts of CO_2_ derived from the C_3_ soil and C_4_ substrate, δ_t_ is the δ^13^C value of the C_t_ (from the total CO_2_), δ_3_ is the δ^13^C value of the C_3_ soil (δ^13^C = −26.36 ~ −0.01‰ and δ^13^C = −24.78 ~ −0.07‰ Cambisol and Fluvisol respectively), and δ_4_ is the δ^13^C value of the C_4_ substrate (δ^13^C = −11.97 ± 0.12‰). Thus, the CO_2_-C produced by the substrate (sucrose) during the incubation could be determined.

The priming effect (or primed soil CO_2_-C) with the addition of sucrose was calculated from: 

4 $$\text{Priming}\,\text{effect}={{\text{C}}}_{{\text{SOM}}\left(\text{substrate}\,\text{added}\right)-}{{\text{C}}}_{{\text{SOM}}\,(\text{without}\, \text{substrate}\,\text{added})}$$

PE was calculated as the difference between SOM-derived CO_2_ from soil substrate added (C_SOM(substrate added)_) and SOM-derived CO_2_ from soil without substrate added (C_SOM(without substrate added)_).

### C retention and loss calculations

The theoretical average C content of each treatment was calculated according to the following equation:

5 $${\text{Theoretical}}\,{\text{C}}\,{\text{content}}={{\text{C}}}_{1}{{\text{W}}}_{1}\,+{{\text{C}}}_{2}{{\text{W}}}_{2}$$

where, *C*_*1*_ is the C content of biochar (g C kg^−1^), *W*_*1*_ is the proportion of biochar mass in the soil-biochar mixture of each treatment, *C*_*2*_ is initial soil C content (g C kg^−1^), and *W*_*2*_ is the proportion of soil in the soil–biochar mixture of each treatment.

The C content of each aggregate fraction was calculated. These values were summed to determine total C retention for each treatment at the end of the experiment according to the following equation (Sheng et al. 2020):

6 $$\text{Total}\,{\text{C}}\,\mathrm{retention }={\sum }_{{\text{i}}=1}^{4}{{\text{C}}}_{{\text{i}}}\times {{\text{P}}}_{{\text{i}}}$$

where, *Ci* is the C content of each aggregate fraction (g C kg^−1^), and *Pi* is the proportion of the whole soil mass represented by each aggregate fraction. The difference between theoretical average C content and the sum of C in the aggregate fractions was defined as C loss during the incubation, calculated according to the following equation:

7 $$\mathrm{Total }\,{\text{C}}\,{\text{loss}}={\text{Theoritical}}\,{\text{C}}\,\mathrm{ content}-\mathrm{Total}\,{\text{C}}\,{\text{retention}}$$

### Synchrotron‐based X-ray CT analysis

Synchrotron‐based X-ray CT scanning combined with imaging was used to determine soil pore structure and total porosity. At the completion of the incubation, soil cores (40 mm diameter and 50 mm length) were assessed using 320 kV X-ray computed tomography in situ (NIKON, United Kingdom) to provide a high-resolution 3D soil pore structure. In each scan, 1250 angular projection images were collected, and each radiograph was averaged over 32 frames. Ring artifacts were minimized during data acquisition, and a 0.5 mm copper filter was used to reduce beam hardening. The software package CT-Pro v.1.0 (Metris X-Tek Systems Ltd., Hertfordshire, United Kingdom), which employs the filtered back-projection algorithm for CT reconstruction, was used to obtain the three-dimensional maps of attenuation coefficients from the two-dimensional angular projections. The three-dimensional images of attenuation coefficients with the isotropic voxel size of 0.0734 mm were then translated into a continuous stack of two-dimensional 16-bit TIFF images (Additional file 1: Fig. S4) using the software VG Studio MAX 1.2.1 (Volume Graphics GmbH, Heidelberg, Germany). The quantitative analysis of pores is mainly using Image J software based on the intervals of each agglomerate, with a size of 400*400*400 pixels within each macroaggregate (Schneider et al. 2012).

### Analysis of 16S rRNA

DNA was extracted from 0.50 g of freeze-dried soil using a Fast DNA Spin Kit (MP Biomedicals, Santa Ana, CA, USA) according to the manufacturer’s protocol. The extracted DNA was dissolved in 50 μl of TE buffer, and the concentrations of DNA were quantified using a Nanodrop 2000 (Thermo Scientific, Willmington, USA). Samples were stored at –80 ˚C before sequencing. The bacterial 16S rRNA gene fragments were amplified using primer sets targeting the V4-V5 variable region. The forward primer is 515F (5’-GTGCCAGCMGCCGCGGTAA-3’) linked with a specific-sample 5-bp barcode sequence at the 5’end of primer, and 806R (5’-GGACTACHVGGG TWTCTAAT-3’) was used as the reverse primer. Each sample was amplified in triplicate, then the three reaction products were pooled and purified using Agincourt Ampure XP beads (Indianapolis, USA). All amplicons were pooled across all samples at equimolar concentrations (20 ng μl^−1^) into a composite sample, and the index sequencing of paired-end 250 bp was performed on an Illumina HiSeq 2000 platform. All the procedures for bacterial and fungal DNA amplification and sequencing were performed by Major bio, Inc. (Shanghai, China).

The raw 16S rRNA gene sequencing reads were demultiplexed, quality-filtered by Trimmomatic, and merged by FLASH with the following criteria: (i) The 300 bp reads were truncated at any site receiving an average quality score of < 20 over a 50 bp sliding window, truncated reads shorter than 50 bp, or containing ambiguous characters were discarded; (ii) Only overlapping sequences longer than 10 bp were assembled according to their overlapped sequence. The maximum mismatch ratio of the overlap region is 0.2. Reads that could not be assembled were discarded; (iii) Samples were distinguished according to the barcode and primers, and the sequence direction was adjusted. Operational taxonomic units (OTUs), with a 97% similarity cut off (Liu et al. 2017), were clustered using UPARSE (version7.1, http://drive5.com/uparse/), and chimeric sequences were identified and removed. The taxonomy of each OTU representative sequence was analyzed by RDP Classifier (http://rdp.cme.msu.edu/) against the 16S rRNA database (e.g., Silva SSU128) using a confidence threshold of 0.7. Equimolar purified amplicons were pooled and paired-end sequenced (2 × 300) on an Illumina MiSeq platform (Illumina, San Diego, USA) according to the standard protocols by Majorbio Bio-Pharm Technology Co. Ltd (Shanghai, China). The amplicon sequence data were deposited in National Centre for Biotechnology Information under the accession number PRJNA 817743.

### Statistical analysis

Shannon index was calculated for bacterial community. For β-diversity analysis, the dissimilarity of bacterial communities was calculated via principal coordinates analyses (PCoA). Distance-based linear model multivariate analysis (distLM) was conducted in distLM forward3 software (Anderson 2003) and used to determine the relative effects of soil variables such as TN, TC, C:N, DOC, MBC, pH, CEC, BD, pore size, porosity, and pore connectivity on soil bacterial community. Two-way orthogonal partial least squares (O2PLS) analysis was performed using the SIMCA-P 14 (Version 14.1.0.2047) to correlate the microbial genera to the dynamics of the substrate mineralization and PE. The Y-matrix was designed as the C dynamics datasets, and the X-matrix was designed as the microbial community data sets (Trygg and Wold 2003). To test the relative importance of environmental variables in driving substrate mineralization and PE, we used a random forest analysis (Liaw and Wiener 2002). Environmental variable validation was done for soil chemical properties (CEC, pH, TN, TC, C:N ratio), physical properties (> 2 mm aggregates, 0.25–2 mm aggregates, < 0.25 mm aggregates, pore connectivity), and biological properties (Network occurrence, co-occurrence pattern of bacteria in-network (eigen values), bacterial diversity, the relative abundance of Firmicutes, Proteobacteria and Actinobacteria). The random forest package was used to estimate the contributions/influences of the above-mentioned variables on C dynamics, including the substrate mineralization and PE. The pathways and drivers of substrate mineralization and PE were investigated by structural equation modeling (SEM), which can determine the direction, magnitude, and effect relationships. The SEM was conducted using AMOS 21.0 to confirm possible causal relationships between abiotic variables and the biotic community on C dynamics. In the SEM, chi-squared values were used to evaluate model fitting, while a non-chi-squared test (*P* > 0.05) indicates a good fit of the model to the data. The analysis of correlation metrics calculated the coefficients of each path. The path in this model was considered significant with a *P* < 0.05. A correlation network for all three different biochar amendmends of Cambisol and Fluvisol was generated to visualize the associations between the diversity of different phyla and the measured environmental variables such as > 2 mm aggregates, 0.25–2 mm aggregates, < 0.25 mm aggregates, pore connectivity CEC, pH, TN, TC, C:N ratio using the Cytoscape. For the construction of networks, the OTUs with relative abundances greater than 0.01% were kept, and the dissimilarity threshold to the maximum value of the KLD matrix and the Spearman's correlation threshold of 0.8 were calculated. For each edge and measure, permutation and bootstrap distributions were computed with 100 iterations. Measure-specific *P*-values were generated as the area of the mean of the permutation distribution under a Gauss curve, computed from the mean and standard deviation of the bootstrap distribution. The *P-*values were adjusted using the Benjamini–Hochberg procedure (Benjamini and Hochberg 1995). Finally, only edges supported by two measures and with adjusted *P*-values below 0.05 were retained. The nodes in the constructed networks represent OTUs, and edges represent strong and significant correlations between OTUs. Network visualizations were conducted using Gephi (Bastian et al. 2009) and Cytoscape 3.5.1 (Shannon et al. 2003). The Network Analyzer tool was used to calculate the network topology parameters. Genera with the highest betweenness centrality scores were considered keystone species (Martín González et al. 2010).

The statistical analysis of all non-microbial data was performed using SPSS 20 (SPSS, Inc., Chicago, IL, USA). One way analysis of variance (one-way ANOVA) was used to analyze the effect of biochar addition. Residuals were checked for normal distribution and homogeneity by Shapiro–Wilk and Levene’s tests, respectively. If conditions were met, the Tukey Post-hoc test was performed to reveal differences between the treatments. All comparisons were made within each sampling date.

## Results

### The substrate derived CO_2_ evolution, and priming effects

**Fig. 1:**
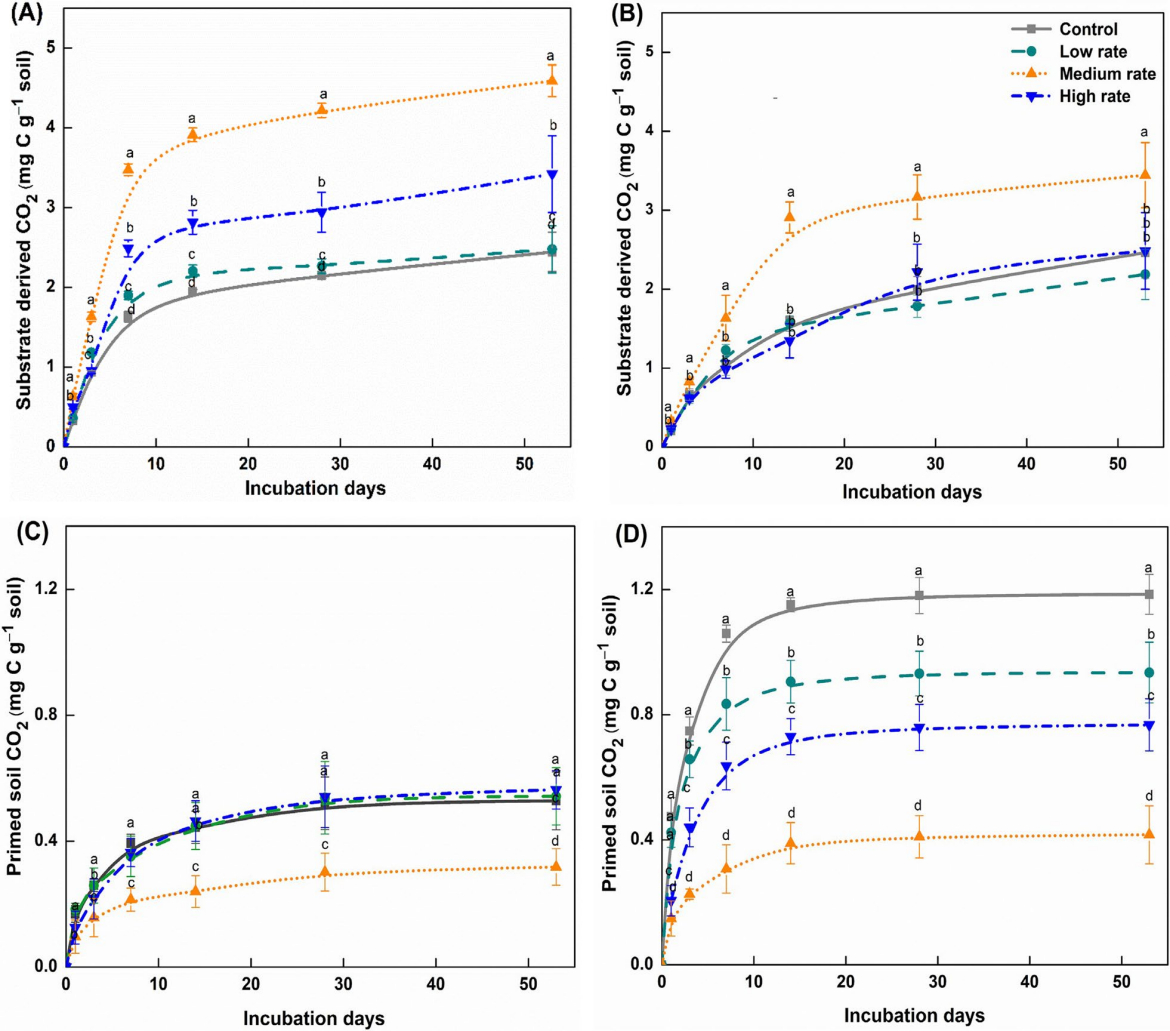
Cumulative sucrose-derived CO_2_ evolved from the Cambisol (**A**), Fluvisol (**B**) and cumulative primed soil CO_2_ evolved from the Cambisol (**C**), Fluvisol (**D**) caused by the various biochar doses: Control (no biochar addition), low rate (25 and 30 Mg ha^−1^), medium rate (50 and 60 Mg ha^−1^) and high rate (75 and 90 Mg ha^−1^), after 53 days of incubation. Error bars represent standard errors of the means (n = 3)

During the 53-day incubation period, higher cumulative mineralization of applied substrate was observed in the Cambisol (2.4–4.5 mg of C g soil^−1^) compared to the Fluvisol (2.1–3.4 mg of C g soil^−1^) (Fig. 1A and B). The application dose of biochar at 50 Mg ha^−1^ in the Cambisol had the highest cumulative substrate-derived C mineralization, followed by 75 Mg ha^−1^. The 25 Mg ha^−1^ biochar application rate was only slightly greater than the unamended Control, but still statistically significant (Fig. 1A). A similar trend was observed in the Fluvisol, with 60 Mg ha^−1^ of biochar presenting with the highest substrate mineralization, but 90 Mg ha^−1^ and 30 Mg ha^−1^ not proving different from the Control (Fig. 1B). Further substrate derived C fluxes were highest on day 7 and day 14 after incubation in Cambisol and Fluvisol, respectively (Additional file 1: Fig S1).

The PE on native SOC mineralization through the addition of sucrose resulted in an immediate (within the first week) increase of soil CO_2_. Cumulative PE reached a peak within 2–3 weeks and, following day 28, negligible PE was detected in both soils (Fig. 1C and D). The cumulative PE on native SOC mineralization following substrate addition was the lowest in the 50–60 Mg ha^−1^ biochar treatment (0.3 and 0.4 mg of C g^−1^ of soil on day 53) in both Cambisol and Fluvisol. In comparison, the cumulative PE was the highest in the Control (1.2 mg of C g^−1^ of soil) on day 53 of the incubation (Fig. 1D). There was a decrease in PE of CO_2_-C by 0.2 and 0.8 mg of C g^−1^ soil in the Cambisol and Fluvisol, respectively, amended with 50–60 Mg ha^−1^ biochar application (Fig. 1C and D).

### Aggregate-associated C storage and losses

The impacts of biochar on aggregate-associated C storage largely mirrored the soil C balance. The retention of soil C in the 50–60 Mg ha^−1^ biochar application rates was 14.2 g C kg^−1^ for the Cambisol and 9.4 g C kg^−1^ for the Fluvisol at the end of the incubation (Additional file 1: Table S1). When examining total C loss (substrate mineralization + SOC priming) during the incubation, the Cambisol and Fluvisol with biochar dose of 75 Mg ha^−1^ lost 12.5 g C kg^−1^ and 12.9 g C kg^−1^ compared to other biochar application doses. In the Fluvisol, soil with 90 Mg ha^−1^ of biochar lost 8.5 g C kg^−1^ soil. Total C retention was highest for both soil types where biochar was applied at 50–60 Mg ha^−1^, and the highest loss of C was found where biochar was applied at 75–90 Mg ha^−1^ biochar.

### Soil physical properties

**Fig. 2:**
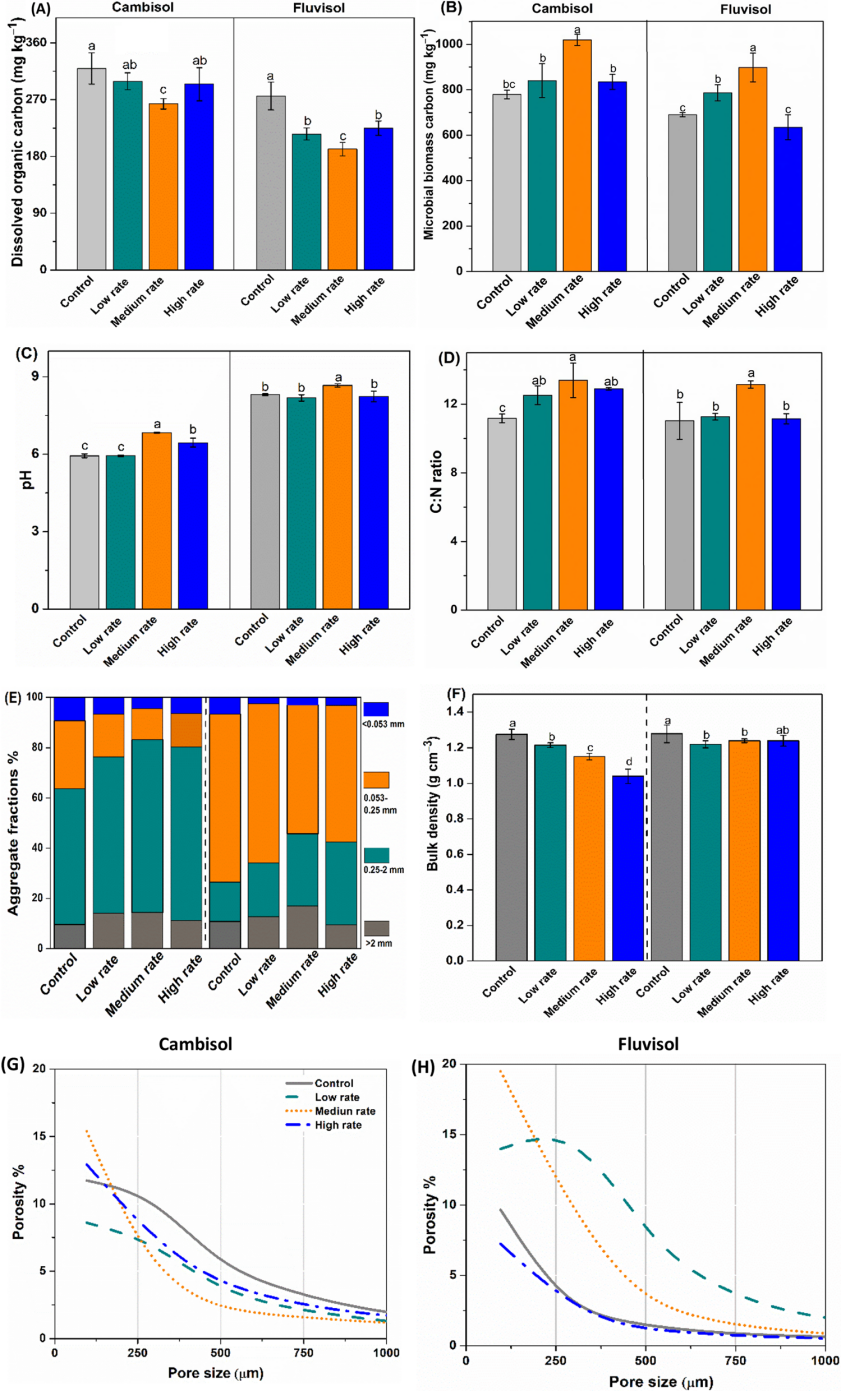
Microbial biomass carbon (**A**) dissolved organic carbon (**B**), pH (**C**), C:N ratio (**D**), soil aggregate size distribution (**E**), bulk density (**F**), and porosity (**G** and **H**) following biochar addition: control, low rate (25, 30 Mg ha^−1^), medium rate (50, 60 Mg ha^−1^), and high rate (75, 90 Mg ha^−1^) doses. Error bars represent standard errors of the means (n = 3)

Sucrose addition to biochar amended soil, after ten years in two field sites, increased soil pH, MBC, C:N ratio and decreased bulk density particularly for the middle additional rate compared to the Control. In contrast, BD and contents of DOC were significantly decreased under middle biochar amendment (Fig. 2). The results showed an increased quantity of soil aggregates in the 0.25–2 mm size class compared to the Control (Fig. 2E). Increased aggregate stability in the > 2 mm and 0.25–2 mm aggregate size was highest when biochar was applied at the middle doses (i.e., 50 and 60 Mg ha^−1^) to the Cambisol and Fluvisol (1.5 and 1.7 fold, respectively). The BD significantly decreased with increasing biochar dose, having a greater effect in the Cambisol than the Fluvisol (Fig. 2F). Synchrotron‐based X‐ray micro‐computed tomography (X-ray μ-CT) indicated that the overall increase of soil porosity occurred in the lower pore size range (< 0.1 mm) with biochar amendments (Fig. 2G and H). Among different biochar rates, the 50 and 60 Mg ha^−1^ biochar amendment of both Cambisol and Fluvisol soils had the highest soil porosity: 15.3% and 19.5%, respectively. The highest pore size distribution was reported from 50 and 60 Mg ha^−1^ biochar addition to the Cambisol and Fluvisol (Fig. 2G and H).

### Soil microbial community structure

**Fig. 3:**
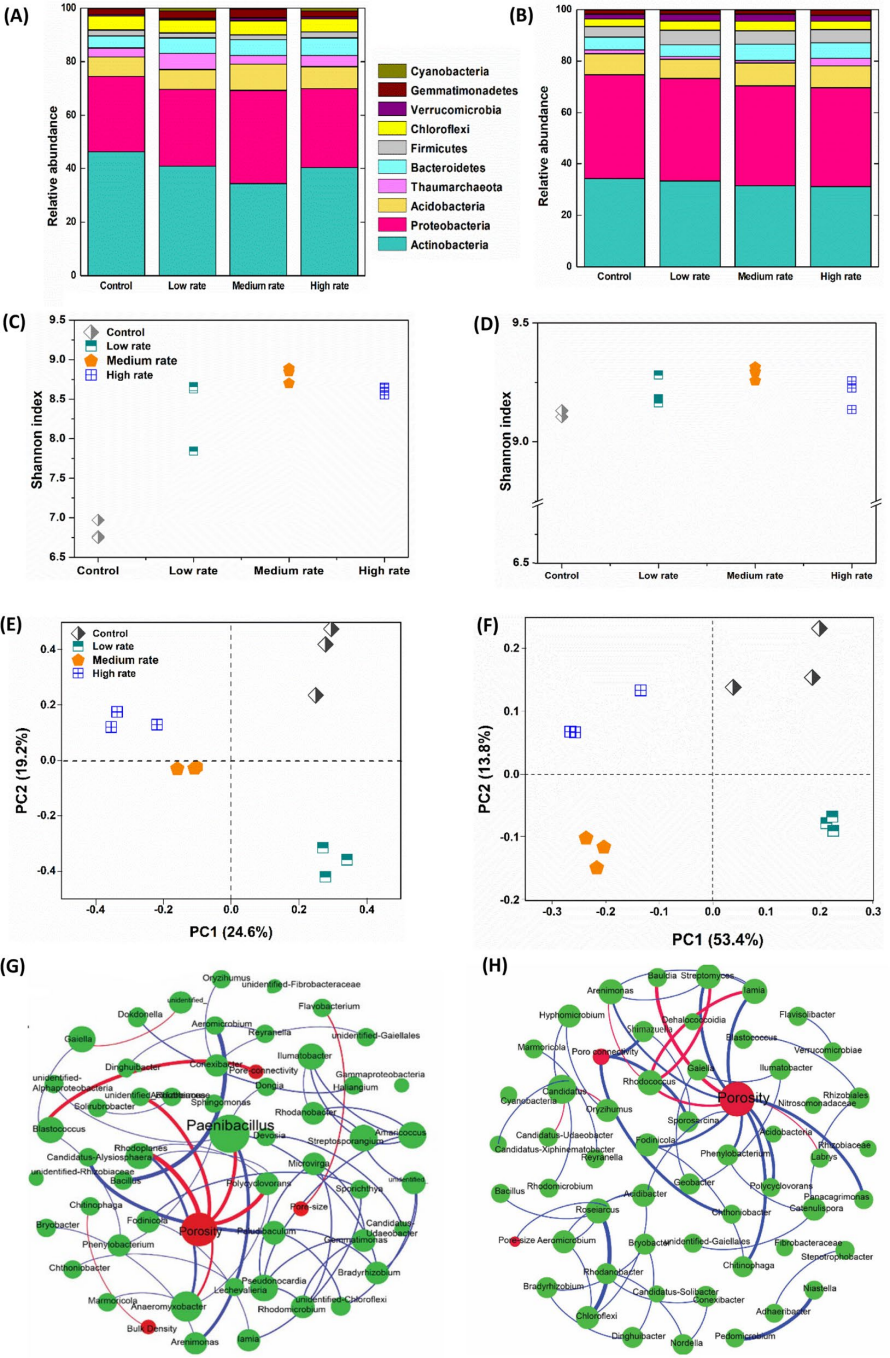
The bacterial community composition (**A**, **B**), alpha diversity by Shannon diversity indices (**C**, **D**), Beta diversity by PCoA analysis (**E**, **F**), and co-occurrence networks of medium biochar dose to Cambisol (**G**) and Fluvisol (**H**). Four biochar doses were assessed: control (nil amendment), low (25, 30 Mg ha^−1^), medium (50, 60 Mg ha^−1^), and high (75, 90 Mg ha^−1^), after 53 days of incubation. For the correlation networks, the relative abundance of the top 200 bacterial OTUs was used. Blue and red lines represent significant positive and negative correlations (*P* < 0.05), respectively. Green circles represent the responsible genera, and red circles represent soil physical factors

The different biochar application doses significantly altered the microbial community structure in the Fluvisol and the Cambisol. The phyla that were most abundant in both soils included Actinobacteria, Proteobacteria, and Acidobacteria. The phylum Proteobacteria showed a significant increase in abundance in the Cambisol at 50 and 60 Mg ha^−1^ biochar application doses, a result not found in the Fluvisol (Fig. 3A and B). The bacterial α-diversity, assessed by the Shannon index, was higher in the 50 and 60 Mg ha^−1^ biochar application rates, while the Control had the lowest diversity in both soils. (Fig. 3C and D). PCoA showed compositional dissimilarities between treatments, with the loadings of PC1 24.6%, PC2 19.2% for Cambisol and PC1 53.4%, and PC2 13.8% for Fluvisol (Fig. 3E and F). The largest variation in bacterial communities occurred following biochar amendment, as indicated by the separation along the first principal coordinate.

The best multivariate distance-based linear modeling (distLM) analysis (Anderson and Legendre 1999) was applied to analyze the contributions of edaphic factors that determined the microbial community, including TN, TC, C:N, DOC, MBC, pH, CEC, BD, pore size, porosity, pore connectivity and > 2 mm aggregate fraction to the microbial community. The soil bacterial community was affected by DOC (18%), MBC (16%), pH (18%), CEC (19%), BD (17%), porosity (19%) and pore connectivity (15%). The bacterial community variations (51%) were explained by soil physical factors (porosity, pore connectivity and bulk density). Soil chemical properties (DOC, pH, and CEC) occupied 55% of the bacterial contribution (Table 1).

**Table 1:** Contributions of edaphic variables to the bacterial community as analyzed by distance-based linear modelling (distLM) analysis

Variable	Bacterial contribution
CEC	0.19**
Porosity	0.19*
pH	0.18**
DOC	0.18*
Bulk density	0.17*
MBC	0.16*
Pore connectivity	0.15*
TN	0.13*
TC	0.12
C:N	0.11
Pore size	0.11

^*^p < 0.05; **p < 0.01

*CEC* cation exchange capacity, *DOC* dissolved organic carbon, *MBC* microbial biomass carbon, *TC* total C, *TN* Total Nitrogen, *C:N* Carbon/Nitrogen ratio

In co-occurrence networks, the application of biochar modified the interactions between bacterial and environmental factors (Fig. 3G, H, Additional file 1: Figs. S2 and S5). Although there were more positive interactions (co-presence) in the bacterial networks of low and high biochar additional rates, interactions between physical properties (porosity, bulk density, pore connectivity and pore size) and keystone taxa were more prominent in the 50–60 Mg ha^−1^ biochar rate compared with other biochar doses (Fig. 3G and H). *Arenimonas, Sphingomonas, Devosia* and *Paenibacillus* were positively correlated with substrate mineralization using O2PLS analysis and the keystone genera cause community stability in co-occurrence networks (Fig. 3G and H, Table 2 and Additional file 1: Fig. S3). *Blastococcus* and *Rhodococcus* were keystone genera in the Control that showed a negative correlation with porosity and a positive correlation with the PE (Fig. 3G and H, Table 2 and Additional file 1: Table S3).

**Table 2:** Two-way orthogonal partial least squares analysis to reveal the core functional genera (with variable influence projection (VIP) > 1.4) involved in C dynamics, including priming effect (PE) and substrate mineralization

Phylum	Genus	VIP value	Priming effect	Substrate mineralization
Proteobacteria	*Arenimonas*	1.63	− 0.55**	0.88***
	*Sphingomonas*	1.56	− 0.78**	0.84**
	*Devosia*	1.52	− 0.71*	0.78***
Actinobacteria	*Blastococcus*	1.62	0.73**	0.66**
	*Rhodococcus*	1.59	0.81**	–
	*Gaiella*	1.58	0.94***	–
Firmicutes	*Paenibacillus*	1.79	− 0.69**	0.86**
	*Shimazuella*	1.46	− 0.73**	0.78*

^*^p < 0.05; **p < 0.01; ***p < 0.001

**Fig. 4:**
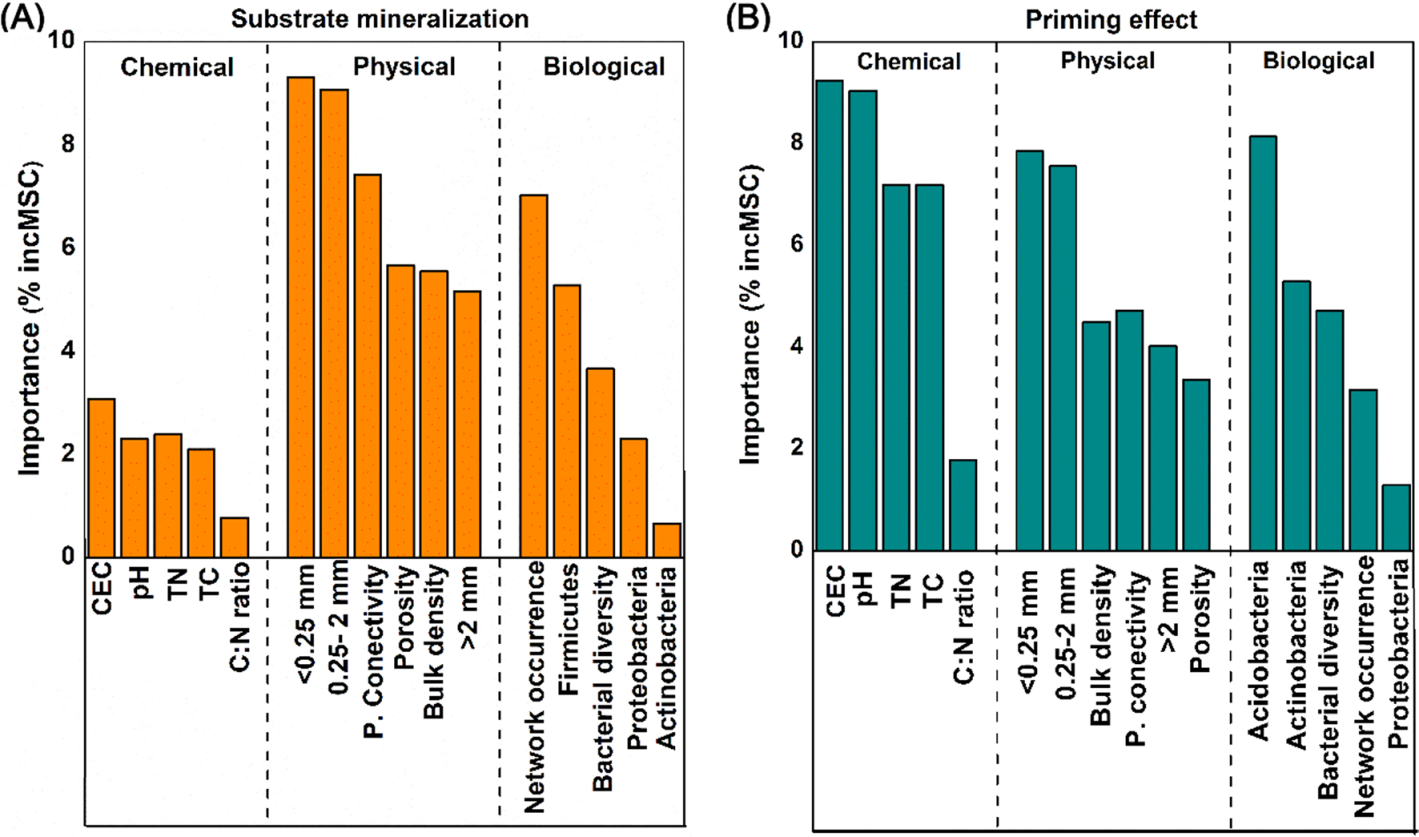
Random forest represents the relative importance of soil physical, chemical and biological variables for substrate mineralization (**A**) and SOC mineralization (**B**). *CEC* cation exchange capacity, *TN* total nitrogen, *TC* total carbon, *C:N* carbon nitrogen ratio, > 2 mm, > 2 mm aggregates; 0.25–2 mm; 0.25–2 mm aggregates, < 0.25 mm; < 0.25 aggregates, P. connectivity, Pore connectivity; Network occurrence pattern represent co-occurrence pattern of bacteria by eigen values; Bacterial diversity; alpha diversity of bacteria

Random forest analysis was used to evaluate the potential predictors of substrate-derived C mineralization and PE on native SOC (including aged biochar). We found that the physical factors (porosity, pore connectivity, BD, > 2 mm aggregate class, 0.25–2 mm aggregate class, and < 0.25 mm aggregate class) were the main determinants of substrate-derived C mineralization (Fig. 4A), whereas chemical factors (TC, TN, CEC, pH, and C:N ratio) contributed more to the PE than physical and biological variables, including substrate-derived C mineralization (Fig. 4A and B). Network co-occurrence patterns and abundance of Firmicutes were the most-likely predictors for substrate-derived C mineralization. The bacterial community composition and bacterial diversity were the most important contributors towards the PE on native SOC. To quantify the relative importance of the different controlling factors on PE and substrate mineralization, an SEM was constructed based on the known relationships between the PE and substrate-derived C mineralization. The effects of substrate application showed a reasonable fit to our hypothesized causal relationships (Additional file 1: Fig. S2). The model accounted for 78% variance of substrate mineralization and 81% variance of the PE. Porosity, pore connectivity, DOC, network co-occurrence, and Firmicutes exerted a dominant direct positive effect on substrate-derived C mineralization. Actinobacteria and DOC were positively associated with PE, whereas the porosity, pore connectivity, and network co-occurrence pattern were negatively linked with the PE.

## Discussion

### Biophysical mechanisms controlling sucrose mineralization

The amendment of soils with biochar more than a decade prior to sampling for the present study showed more rapid mineralization of applied sucrose (Fig. 1a). It was proposed that substrate mineralization is mainly regulated by biochar-induced changes in soil physicochemical and biological properties, e.g., pH, microbial activity, porosity, aggregation and MBC (Hamer et al. 2004; Singh and Cowie 2014; Watzinger et al. 2014). Similar to our findings, it was reported that biochar amendment after four years increased soil pH, MBC and C:N ratio and decreased bulk density, particularly for the 40 Mg ha^−1^ biochar dose compared to the Control (Zheng et al. 2016). However, recent studies showed that biochar induced changes, such as the improved soil aggregation and porosity could be found in the fields several years after addition (Jones et al. 2012; Liu et al. 2017; Wang et al. 2017). Similarly, the medium application rates (50–60 Mg ha^−1^) resulted in the greatest increase in porosity and aggregation (Fig. 2E, G, H). Further, it was consistent with previous studies showing that biochar amendment to soil increases in µ-CT porosity, pore connectivity and decreases soil bulk density (Herath et al. 2013; Brewer et al. 2014; Quin et al. 2014; Liu et al. 2017). Indeed, Quin et al. (2014) amended biochar to soil at 5% (w/w) (consistent with around 50–60 Mg ha^−1^ in the current study) and showed the greatest increase in porosity and pore connectivity in two different soils (*Vertisol* and *Ferralsol*).

Accordingly, the high porosity (< 0.1 mm) (as reported under the 50–60 Mg ha^−1^ biochar treatments) directly reduced physical constraints and thus (i) promoted microbial accessibility to sucrose, and (ii) increased activity and diversity of microbial community. Greater pore connectivity of soils facilitates the movement of gases (i.e., oxygen), water, soluble organic substrates and nutrients, which can promote microbial activity, diversity and consequently facilitate C mineralization (Ruamps et al. 2011; Ananyeva et al. 2013). The observed higher cumulative substrate mineralization was consistent with previous studies, potentially attributed to the activation of the dormant microbial communities starved of labile C (Chotte et al. 1998; Hamer et al. 2004; Hamer and Marschner 2005). It was reported that the high porosity provides habitat for soil microbial colonization (Luo et al. 2013) and modulates their interactions with C/nutrients that can be adsorbed by biochar (Hockaday et al. 2006; Herath et al. 2013). An increase in soil bacterial alpha diversity (Shannon) in biochar amended soils (50–60 Mg ha^−1^) was also observed (Fig. 3C and D), suggesting greater sucrose utilization (Barret et al. 2011; Carbonetto et al. 2014; Chen et al. 2021). Similarly, it was reported that the presence of labile C and biochar resulted in greater microbial diversity and promoted greater substrate mineralization (Anderson et al. 2011).

Among the linkages of the bacterial networks, co-presence (positive interactions) was increased under biochar amendment at 50–60 Mg ha^−1^, suggesting mutually beneficial interactions of bacteria for substrate acquisition (Herath et al. 2013; Brewer et al. 2014; Liu et al. 2017). Chen et al. (2019) found that biochar-induced competition within microbial groups stimulated soil microbial diversity and thus reduced C mineralization. However, interactions between 25–30 and 75–90 Mg ha^−1^ biochar amended soil did not represent strong relationship with physical properties in both soils, supporting that middle application rate (50–60 Mg ha^−1^) of biochar strongly influenced physiochemical properties of the soils while shifting the microbial community (Fig. 3G, H and Additional file 1: Fig. S5). It was reported that among the physical properties, biochar application reduced bulk densities by 29% and increased porosity by 59% (Singh et al. 2022). Different aspects of soil structure also improved in response to amendments including biochar (Blanco-Canqui 2017), and resulted in changes in microbial composition, abundance, and activities (Lehmann et al. 2011) by providing favourable environments with aeration, water, and nutrients (Ameloot et al. 2014). Because of microbial shifting, key soil processes such as C mineralization could be altered by the biochar additional rate (Ullah et al. 2020). The inconsistent results might be due to differences in types of biochar, soils and aging effects that modulated opposite traits of dominant microbiotas. Drivers of inconsistencies in responses are various, including heterogeneity among experiments related to soil types and rates of biochar application (ranging from 5 to 150 t ha^−1^), properties of biochar as a function of feedstock (Atkinson 2018). Still, the comprehensive understanding of microbial interactions underlying C dynamics shaped by changes in biochar-induced soil physicochemical properties remains largely unexplored.

### Insight into physicochemical mechanisms underlying biochar caused SOC priming

Since the priming of SOC could negate the biochar C sequestration potential in soil, trade-offs between new input C stabilization and SOC priming need to be better understood and quantified. There is a paucity of knowledge on substrate-induced priming of SOC from long-term biochar field trials. The current study showed that substrate (sucrose, as a proxy for rhizodeposits in this study) addition to soil stimulated mineralization of native SOC, causing positive priming (Fig. 1C and D). Interestingly, we found an opposite trend of PE with biochar application, compared to sucrose mineralization. As indicated by the random forest analysis, modification of soil properties (e.g., pH, MBC, TN, and TC), and the modulation of microbial communities (Proteobacteria, and Firmicutes) by biochar showed a combined effect on the PE following the addition of sucrose (Fig. 4). It was documented that several factors are responsible for priming in long-term biochar incorporated soils like chemical structure of SOC, microbial community composition, and soil aggregation (Herath et al. 2015; Martinsen et al. 2015; Wang et al. 2016). Biochar applied at 50–60 Mg ha^−1^ to field sites more than a decade ago showed suppressed sucrose-induced PE by 2 to 3 folds than that of the Control (Fig. 1C and D). This could have occurred as a result of the high pH, MBC, C:N ratio and modified microbial community in the middle biochar rate soil. Most studies suggested that biochar could affect the abundance of microorganisms due to its direct changes on the physicochemical properties of the soil (Lehmann et al. 2011; Ameloot et al. 2014; Jaafar et al. 2014). Some previous studies reported that the microbial communities and bacterial diversity in the soil change differently with long-term biochar application (Khodadad et al. 2011). As such, PE is complicated as a result of the ratio between “r-strategist” and “K-strategist”, which depends on pH, nutrient status, substrates, (Rasul et al. 2022). Interestingly, dominant microbial taxa in middle dose biochar falling into the “r-strategist” are better adapted for rapid response to new applied C sources than native SOC leading low SOC mineralization.

Other explanations may include middle biochar application maintained a greater MBC, aggregation and lower DOC, yet incorporation of C into physically protected soil fractions was possible. This observation may hint at processes of SOC stabilization can occur via processes such as improved aggregation, adsorption, or compartmentalization. The main mechanisms of the lower PE induced by biochar involved soil aggregate formation, which physically protects SOC against microbial mineralization (Du et al. 2017; Weng et al. 2017; Zheng et al. 2018). Previous studies have demonstrated that biochar stimulates aggregate formation, which limits microbial access to SOC and thus causes lower mineralization of native SOC (Yu et al. 2012; Wang et al. 2017). In another study, biochar enhanced aggregation in finer-textured soils was associated with an increase in physically protected C incorporated into macro-aggregates, including both > 2 mm (large macroaggregates) and 0.25–2 mm (small macroaggregates) (Wang et al. 2017).

In contrast to the middle application dose, we observed higher SOC loss in the 75–90 Mg ha^−1^ biochar treatment, which had core genera such as *Streptomyces* (Additional file 1: Table S3) and smaller effects by aggregation (Additional file 1: Table S1). It was reported that *Streptomyces* bacteria are well adapted to complex ecosystems, composed of complex C substrates where they grow mycelium with multiple hyphae to search SOC (Antido and Climacosa 2022). Moreover, the absence of a substantial aggregate structure in soil could result in greater accessibility of SOC, nutrients and oxygen to microbes, which is likely to stimulate SOC mineralization (Dungait et al. 2012).

Several other mechanisms may be involved in the biochar-induced changes in soil properties that result in PE. The development of organo-mineral complexes through interactions with negatively charged surface functional groups on SOC (e.g., R–COO–) and soil minerals (e.g., Al–O–, Fe–O–, and Si–O–) describes mechanisms involved in biochar induced C stabilization (Joseph et al. 2010; Weng et al. 2018). These interfacial reactions enhance the oxidation resistance of biochar particles leading to the lower PE induced by new substrates, such as rhizodeposits (Yang et al. 2016; Weng et al. 2018). Based on the literature, the “aging” of biochar results in the formation of organic functional groups on biochar surfaces (Cheng et al. 2006; Lützow et al. 2006). Thus, this might enhance biochar interactions with native SOC and clay minerals e.g. through ligand exchange or cation bridging mechanisms (Lützow et al. 2006). In summary, biochar applied at 50–60 Mg ha^−1^ lowered substrate-induced SOC priming, resulting in the modified microbial community due to changes of soil physicochemical properties such as pH, MBC, CEC and aggregation that protect SOC from microbial access.

### Involvement of core taxa on sucrose mineralization and SOC priming

Random forest analysis showed that the core bacteria such as Firmicutes and Proteobacteria revealed by the co-occurrence network (eigen values) had the greatest effects on sucrose mineralization (Fig. 4). Similarly, O2PLS analysis indicated the significance of the soil bacterial phyla Proteobacteria and Firmicutes in substrate mineralization (Table 2), especially the genera *Arenimonas, Sphingomonas* (γ-Proteobacteria), *Paenibacillus* and *Shimazuella* (Firmicutes). Dominance of these fast growth genera (r-strategists) might explain larger sucrose mineralization (Table 2), as these r-strategists are quickly adapted to respond to newly available C sources (Kuzyakov and Gavrichkova 2010; Luo et al. 2011). These results are consistent with other reports, where the genera *Sphingomonas* and *Devosia* (Proteobacteria) have been reported to dominate by utilizing labile C resource as fast-growing r-strategists (Fierer et al. 2007; Sun et al. 2022). Furthermore, it was reported that the genera *Arenimonas* and *Spingomonas* can utilize organic acids, modulate C and nutrient intake according to their metabolic needs, facilitating labile C mineralization (Carbonetto et al. 2014; Makk et al. 2015; Schostag et al. 2019).

According to O2PLS and Random Forest analysis, there are several genera (e.g., *Blastococcus* and *Arenimonas*) utilizing both sucrose and SOC. The stimulation of SOC mineralization after substrate addition to the biochar amended soil has been attributed to co-metabolic mineralization of SOC by the microbial enzymes, which were produced to utilize the labile C in soil (Singh and Cowie 2014; Cheng et al. 2017). This supports our findings of fast-growing genera, e.g., *Arenimonas, Spingomonas* which were positively correlated with PE. Further, less priming accompanied with greater substrate mineralization may occur due to substrate-switching among those genera by the preferential use of a labile C source over a more refractory one (Zimmerman and Ouyang 2019).

The phylum Actinobacteria is considered to contain many representative taxa known to degrade recalcitrant forms of C (Barret et al. 2011). For example, *Blastococcus, Gaillia* and *Rhodococcus* (affiliated to Actinobacteria) can act as regulators of native SOC mineralization with the highest variable influence projection (VIP) having a positive correlation (Table 2). Actinobacteria have a high affinity for both labile C and complex C substrates, and their ability to grow like soil fungi makes it possible to explore the soil in search of C sources (McCarthy and Williams 1992). For instance, Actinobacteria can access C/nutrients using branched filaments in oligotrophic conditions. Moreover, Actinobacteria are generally enriched in enzymes (e.g., glycoside hydrolases) responsible for degrading cellulose, starch and xylan, facilitating recalcitrant C decomposition (Pold et al. 2016). Soils with biochar applied at 50–60 Mg ha^−1^ had a lower abundance of Actinobacteria (Fig. 3), therefore partially explaining the lower priming of SOC following substrate addition (Fig. 1).

### Environmental implications

The potential of utilizing biochar to store C in the soil has received considerable research attention in recent years as part of efforts to develop climate-smart agricultural practices (Weng et al. 2017). The impact of biochar on soil physicochemical and biological properties is vital to the C dynamics (e.g., direction, magnitude and duration of biochar-induced PE) in biochar amended soil. We therefore fully assessed soil physicochemical and biological changes in the soils amended with biochar a decade prior to the present study. Here, we showed the highest sucrose mineralization as well as the lowest SOC priming after amendment of biochar at the medium rate, suggesting the best dose for biochar application to achieve greatest C sequestration effect. Less PE observed in medium application of biochar suggests less native SOC loss, whilst greater mineralization of sucrose implies the increased turnover of new added substrate. Also, more C provision for microbial use may enhance SOC via metabolites and necromass.

**Fig. 5:**
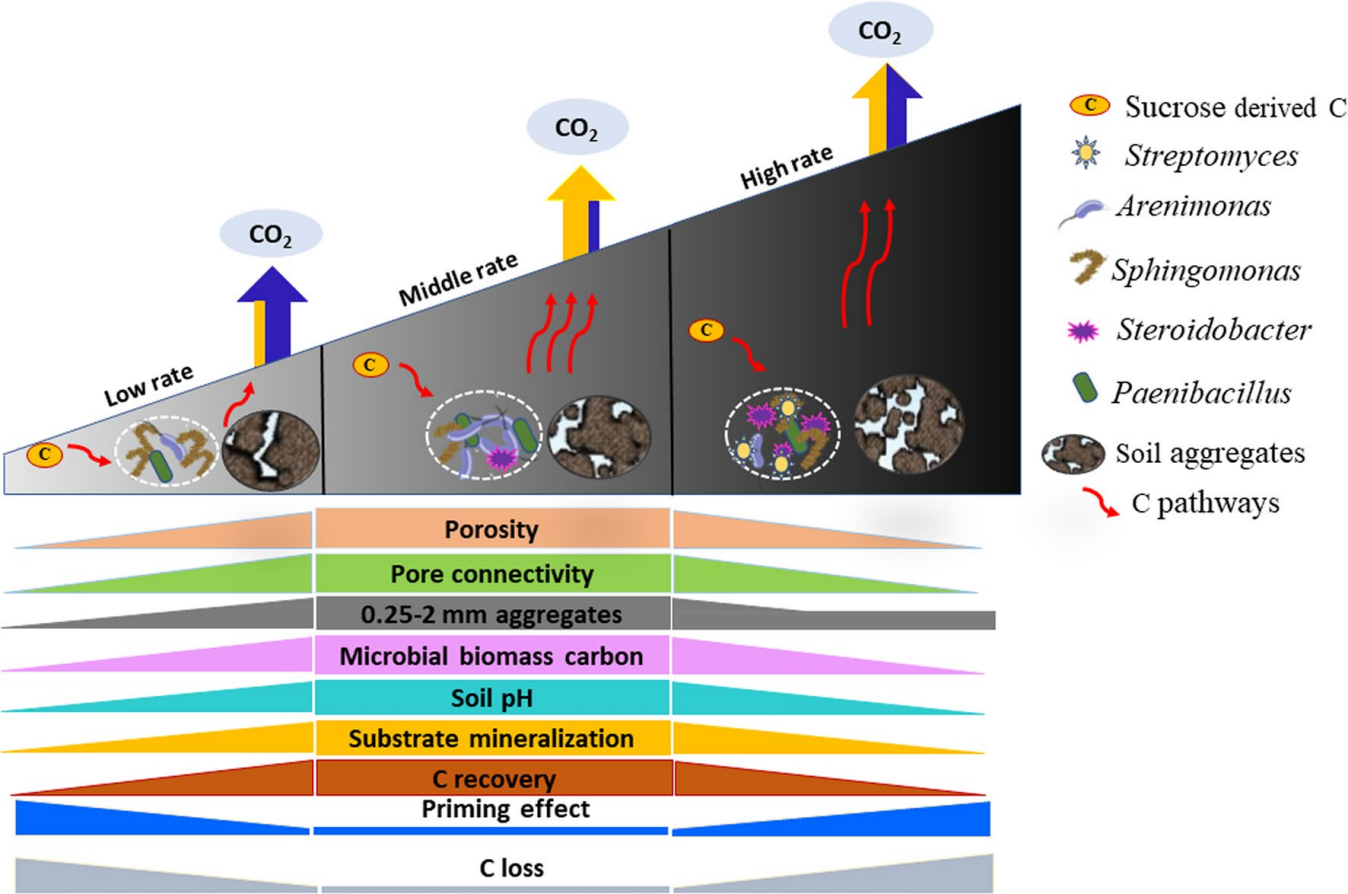
Conceptual diagram of C dynamics via interaction of physicochemical properties (porosity, connectivity) that resulted in higher microbial diversity and accessibility, thus larger new C input mineralization, but offset by lower SOC priming due to the protection provided by enhanced aggregation

It is critical to disentangle the mechanisms (physical, chemical and biological) underpinning the long-term biochar induced C dynamics as these determine both SOC turnover and biochar C sequestration potential in soil. Our study highlights non-biochar C dynamics and provides evidence of how biochar affects C mineralization via soil abiotic and biotic properties (Fig. 5). Various rates of biochar application into the soil affects physiochemical properties differently, thereby modifying the core microbiome in soil towards r-strategist or K-strategist. Thereby the application of middle dose biochar enhanced substrate accessibility to microorganisms, particularly r-strategists, via improved soil porosity and pore connectivity. On the other hand, priming of SOC following sucrose addition was lower in biochar amended soil, with the SOC protected through changes to physicochemical properties such as CEC and aggregates.

## Conclusions

This study investigated the legacy effects of biochar over ten years after application to two managed field trials. The purpose of the study was to evaluate long-term biochar effects, including control of SOC stocks, physicochemical and biological properties. We found that soils amended with biochar at the rate of 50–60 Mg ha^−1^ (medium application rate) 10 years prior to this study showed increased mineralization of applied sucrose (1.9-fold in Cambisol and 1.4-fold in Fluvisol) compared to the Control. This was mainly due to improved soil porosity and connectivity that enhanced microbial accessibility to the labile C. The shift in the bacterial community dominated by fast-growing microorganisms (e.g., *Arenimonas* and *Sphingomonas*) suggests r-strategists benefited from this higher porosity and accessibility to labile C. Biochar application at 50–60 Mg ha^−1^ significantly minimized SOC priming (0.6-fold in Cambisol and 0.3-fold in Fluvisol compared to Control), making it the optimal dose for maximizing biochar's potential for enhanced C sequestration. The promoted aggregation by biochar was associated with the improved protection of SOC. We showed the dominant governing mechanisms of mineralization of substrate (sucrose) and native SOC are biophysical and physicochemical, we also proved that the legacy effects of biochar continue at least a decade after application in comparison to non-biochar C dynamics.

### Supplementary Information

**Additional file 1: Table S1.** Carbon retention and C loss based on theoretical average C content of the treatments (using initial soil and biochar C content) after 53 days of incubation in two soil types (Cambisol, Fluvisol), low rate (25 and 30 Mg ha^-1^), medium rate (50 and 60 Mg ha^-1^), and high rate (75 and 90 Mg ha^-1^), at varying application rates (doses). **Table S3.** Topological features of the networks. **Table S4.** Keystone taxa of networks. **Table S5.** Pore connectivity after 53 days incubation period of Cambisol and Fluvisol caused by the different biochar additional rates: (1) soil without biochar (control) (2) low rate (25 and 30 Mg ha^-1^), medium rate (50 and 60 Mg ha^-1^), and high rate (75 and 90 Mg ha^-1^). Unit of the pore connectivity is Eular number. **Table S6.** Physical and chemical properties of the biochar used in field trial in United Kingdom. **Table S7.** Properties of biochar before its application in the field experiment in China (Dong et al. 2017). **Fig. S1.** Substrate derived CO2 fluxes in Cambisol soil (a) and Fluvisol soil (b) soil over incubation period with the addition of substrate (53 days): Soil only (Control), low rate (25 and 30 Mg ha^-1^), medium rate (50 and 60 Mg ha^-1^), and high rate (75 and 90 Mg ha^-1^). Error bars represent standard errors of the means (n= 3). **Fig. S2.** A structural equation model (SEM) checks the method's description, what each number means used to assess multivariate effects on the priming effect and sucrose mineralization (CO2 efflux). Effects of soil porosity, pore connectivity, DOC (dissolved organic carbon), bacterial diversity, Network co-occurrence, Actinobacteria, and Firmicutes on the priming effect and substrate-C mineralization. The priming effect represents the by CO⁠2 emission from native SOC priming, and substrate-C mineralization represents the CO⁠2 emission from added substrate mineralization. The solid blue lines indicate positive path coefficients and dashed red lines indicate negative path coefficients; R⁠2 values represent the proportion of variance explained for each endogenous variable. **Fig. S3.** Correlation coefficients between the most abundance genera, soil properties under biochar-amended treatments. Abbreviations: MBC; Microbial biomass carbon, BD; Bulk density, AG1;>2mm aggregates, AG2;0.25-2mm aggregates, AG3;<0.25mm aggregates, C/N; Carbon Nitrogen ratio, DOC; Dissolved organic carbon, C; Total carbon, N; Total N, CEC; Cation exchange capacity. Leave only which affect strong, >0.5. **Fig. S4.** Representative 2-D images of macroaggregates for the different Biochar treatments: (A) Control, (B) biochar low addition, (C) biochar medium addition, (D) biochar high addition for Cambisol soil. (E) Control, (F) biochar low addition, (G) biochar medium addition, (H) biochar high addition for Fluvisol soil. The quantification of pore characteristics was based on the selected square. **Fig. S5.** Bacteria co-occurrence network and interaction with physiochemical properties (A,B) biochar low addition, (C,D) biochar high addition for Cambisol soil and Fluvisol soil. The quantification of pore characteristics was based on the selected square.

## Data Availability

All data gathered or analyzed in this study are included in the article.
