# Supplementary material for: Distinct biophysical and chemical mechanisms governing sucrose mineralization and soil organic carbon priming in biochar amended soils: evidence from 10 years of field studies
Source: Biochar. 2024 May 22;6(1):52. doi: 10.1007/s42773-024-00327-0 (PMC11111575; doi:10.1007/s42773-024-00327-0)
Supplement: Supplementary file 1 — Additional file 1: Table S1. Carbon retention and C loss based on theoretical average C content of the treatments (using initial soil and biochar C content) after 53 days of incubation in two soil types (Cambisol, Fluvisol), low rate (25 and 30 Mg ha-1), medium rate (50 and 60 Mg ha-1), and high rate (75 and 90 Mg ha-1), at varying application rates (doses). Table S3. Topological features of the networks. Table S4. Keystone taxa of networks. Table S5. Pore connectivity after 53 days incubation period of Cambisol and Fluvisol caused by the different biochar additional rates: (1) soil without biochar (control) (2) low rate (25 and 30 Mg ha-1), medium rate (50 and 60 Mg ha-1), and high rate (75 and 90 Mg ha-1). Unit of the pore connectivity is Eular number. Table S6. Physical and chemical properties of the biochar used in field trial in United Kingdom. Table S7. Properties of biochar before its application in the field experiment in China (Dong et al. 2017). Fig. S1. Substrate derived CO2 fluxes in Cambisol soil (a) and Fluvisol soil (b) soil over incubation period with the addition of substrate (53 days): Soil only (Control), low rate (25 and 30 Mg ha-1), medium rate (50 and 60 Mg ha-1), and high rate (75 and 90 Mg ha-1). Error bars represent standard errors of the means (n= 3). Fig. S2. A structural equation model (SEM) checks the method's description, what each number means used to assess multivariate effects on the priming effect and sucrose mineralization (CO2 efflux). Effects of soil porosity, pore connectivity, DOC (dissolved organic carbon), bacterial diversity, Network co-occurrence, Actinobacteria, and Firmicutes on the priming effect and substrate-C mineralization. The priming effect represents the by CO⁠2 emission from native SOC priming, and substrate-C mineralization represents the CO⁠2 emission from added substrate mineralization. The solid blue lines indicate positive path coefficients and dashed red lines indicate negative path coefficients; R⁠2 values [file 42773_2024_327_MOESM1_ESM.docx]

**Additional information**

**Distinct biophysical and chemical mechanisms governing sucrose mineralization and soil organic carbon priming in biochar amended soils: evidence from 10-years old field studies**

Haoli Zhang^a^*^#^*, Tao Ma^b^*^#^*, Lili Wang^c^, Xiuling Yu^d^, Xiaorong Zhao^a^, Weida Gao^a^, Lukas Van Zwieten**^f^**, Bhupinder Pal Singh^g^, Guitong Li^a^, Qimei Lin^a^, David R. Chadwick^e^, Shenggao Lu**^d^**, Jianming Xu^d^, Yu Luo^d^, David L. Jones^e,g^ , Peduruhewa H. Jeewani^d,e*^

**Table S1**. Carbon retention and C loss based on theoretical average C content of the treatments (using initial soil and biochar C content) after 53 days of incubation in two soil types (Cambisol, Fluvisol), low rate (25 and 30 Mg ha^-1^), medium rate (50 and 60 Mg ha^-1^), and high rate (75 and 90 Mg ha^-1^), at varying application rates (doses).

|  | **C retention** | | | | | | **C loss** | | | |
| --- | --- | --- | --- | --- | --- | --- | --- | --- | --- | --- |
|  | | | **Cambisol** | | **Fluvisol** | | **Cambisol** | | **Fluvisol** | |
| Low rate | | | 8.48 | | 5.72 | | 10.35 | | 5.82 | |
| Medium rate | | | 14.23 | | 9.40 | | 12.12 | | 8.52 | |
| High rate | | | 12.15 | | 7.00 | | 24.63 | | 19.93 | |

**Table S3:** Topological features of the networks

| **Topographical properties** |  | **Cambisol** |  |  | **Fluvisol** |  |
| --- | --- | --- | --- | --- | --- | --- |
| Biochar additional rates | Low | Medium | High | Low | Medium | High |
| Nodes | 72 | 56 | 42 | 42 | 55 | 42 |
| Edges | 69 | 53 | 29 | 30 | 53 | 28 |
| Modularity | 0.39 | 0.59 | 0.14 | 0.58 | 0.26 | 0.49 |
| Clustering coefficient | 0.17 | 0.21 | 0.05 | 0.06 | 0.2 | 0.41 |
| Average path length | 1.92 | 1.09 | 0.09 | 1.16 | 1.02 | 1.097 |
| Copresence (%) | 98.55 | 79.25 | 72.67 | 76.67 | 81.48 | 71.43 |
| Mutual-exclusion (%) | 1.45 | 20.75 | 27.33 | 23.3 | 18.52 | 28.57 |

**Table S4:** Keystone taxa of networks

| **Biochar additional rate** | **Cambisol** | **Fluvisol** |
| --- | --- | --- |
| **Low** | *Blastococcus* | *Pedomicrobium* |
|  | *Marmoricola* | *Rhodo coccus* |
|  |  |  |
| **Medium** | *Paenibacillus* | *Shimazuella* |
|  | *Conexibacter* | *Arenimonas* |
|  |  |  |
| **High** | *Lobrys* | *Steroidobacter* |
|  | *Reyranell* | *Streptomyces* |
|  |  |  |

**Table S5:** Pore connectivity after 53 days incubation period of Cambisol and Fluvisol caused by the different biochar additional rates: (1) soil without biochar (control) (2) low rate (25 and 30 Mg ha^-1^), medium rate (50 and 60 Mg ha^-1^), and high rate (75 and 90 Mg ha^-1^). Unit of the pore connectivity is Eular number.

|  | **Cambisol** | **Fluvisol** |
| --- | --- | --- |
| **Control** | 21730 | 32020 |
| **Low rate** | -12674 | -71723 |
| **Medium rate** | -86968 | -91266 |
| **High rate** | -86549 | -20807 |

**Table S6**. Physical and chemical properties of the biochar used in field trial in United Kingdom.

| **Properties** | **Biochar** |
| --- | --- |
| Dry bulk density (g cm^−3^) | 0.20 ± 0.01 |
| Moisture content (g kg^−1^) | 35 ± 7 |
| Soil microbial biomass-C (g kg^−1^) | <0.01 |
| pH | 9.8 ± 0.2 |
| Electrical conductivity (μS cm^−1^) | 1133 ± 244 |
| Total C (g kg^−1^) | 461 ± 10 |
| Total N (g kg^−1^) | 6.8 ± 0.1 |
| C-to-N ratio | 67± 2 |
| Water holding capacity (g kg^−1^) | 977 ± 34 |
| Specific surface area (m^2^ g^−1^) | 29.0 ± 4.3 |
| Extractable NO_3_^−^ (mg N kg^−1^) | 0.1 ± 0.0 |
| Extractable NH_4_^+^ (mg N kg^−1^) | <0.1 |
| Available P (mg P kg^−1^) | 12 ± 1 |
|  |  |

Values represent means ± SEM (*n* = 3) and are expressed on a dry weight basis.

**Table S7**

| **Table. S7** Properties of biochar before its application in the field experiment in China (Dong et al. 2017) | | | |
| --- | --- | --- | --- |
| Properties | Biochar |  |  |
| Organic carbon, g kg^−1^ | 491 ± 3 | |  |
| Total nitrogen, g kg^−1^ | 12.2 ± 0.8 | |  |
| SOC/N | 40.2 | |  |
| Inorganic carbon, g kg^−1^ | 10.01 | |  |
| Ash, g kg^−1^ | 360 ± 3 | |  |
| pH | 10.64 ± 0.01 | |  |
| EC, mS cm^−1^ | 1.02 ± 0.05 | |  |
| CEC, cmol(+) kg^−1^ | 12.5 ± 0.1 | |  |
| Particle size | 0.5–5 mm | |  |
| Surface area (m^2^ g^−1^) | 15.68 | |  |

**Fig. S1**

B

A


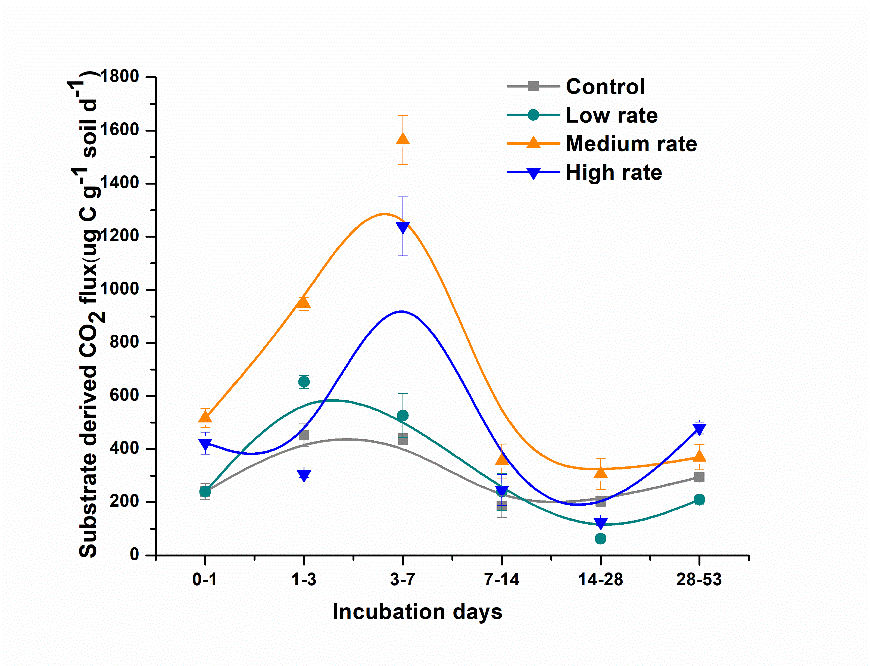

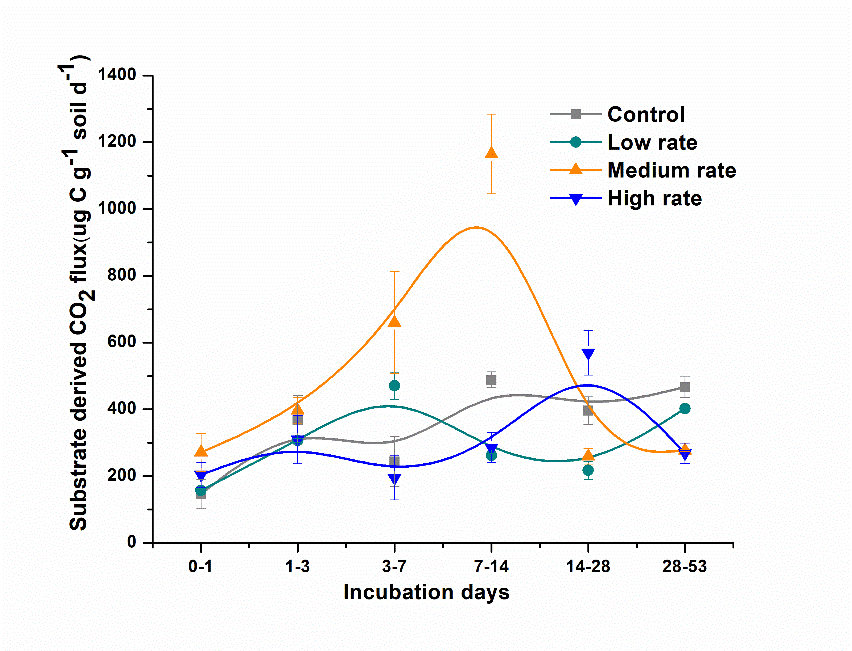


**Fig. S1**: Substrate derived CO_2_ fluxes in Cambisol soil (a) and Fluvisol soil (b) soil over incubation period with the addition of substrate (53 days): Soil only (Control), low rate (25 and 30 Mg ha^-1^), medium rate (50 and 60 Mg ha^-1^), and high rate (75 and 90 Mg ha^-1^). Error bars represent standard errors of the means (n= 3).

**Fig. S2**


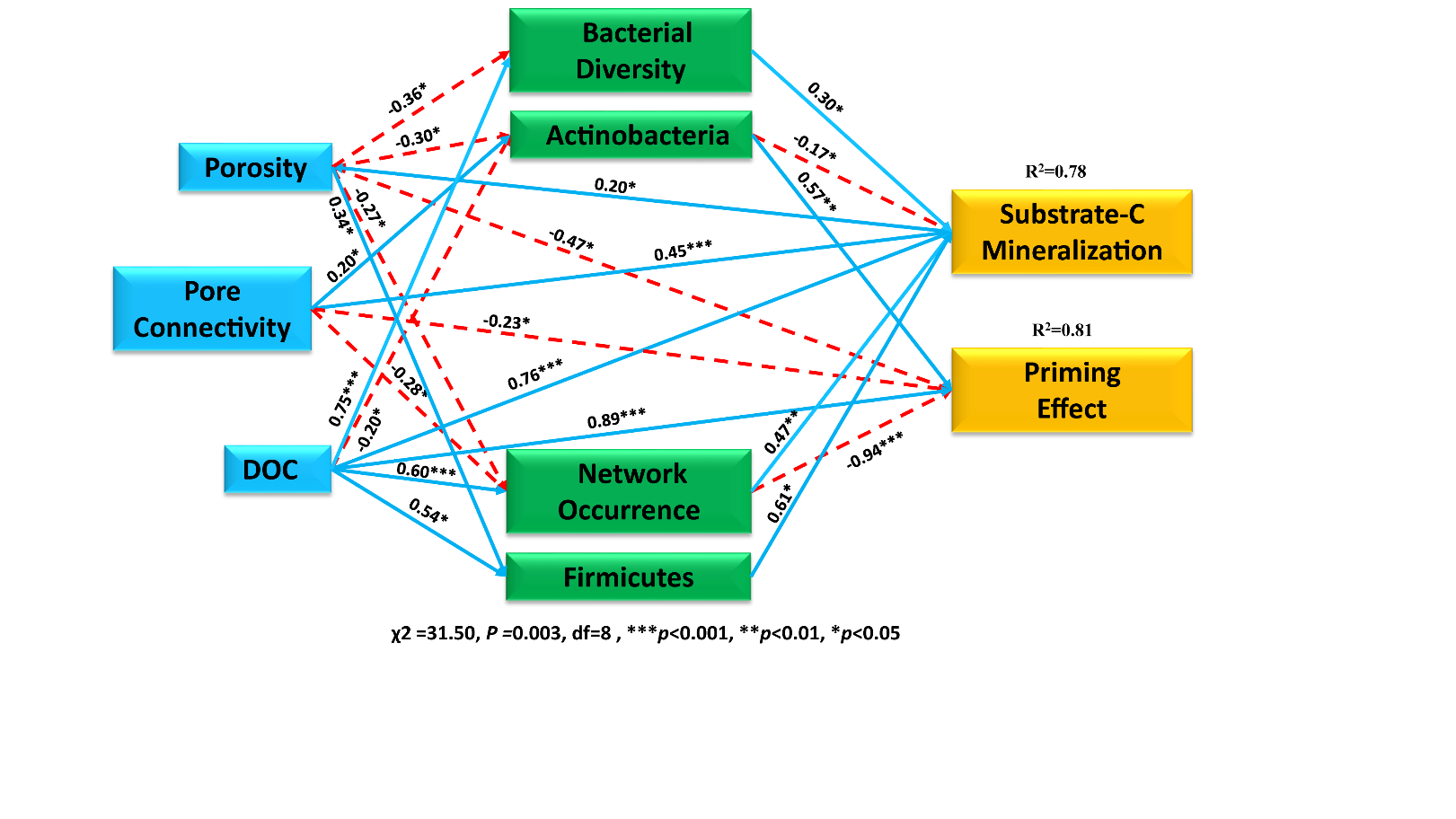


**Fig. S2**. A structural equation model (SEM) checks the method's description, what each number means used to assess multivariate effects on the priming effect and sucrose mineralization (CO_2_ efflux). Effects of soil porosity, pore connectivity, DOC (dissolved organic carbon), bacterial diversity, Network co-occurrence, Actinobacteria, and Firmicutes on the priming effect and substrate-C mineralization. The priming effect represents the by CO_⁠2_ emission from native SOC priming, and substrate-C mineralization represents the CO_⁠2_ emission from added substrate mineralization. The solid blue lines indicate positive path coefficients and dashed red lines indicate negative path coefficients; R⁠^2^ values represent the proportion of variance explained for each endogenous variable.

**Fig. S3**


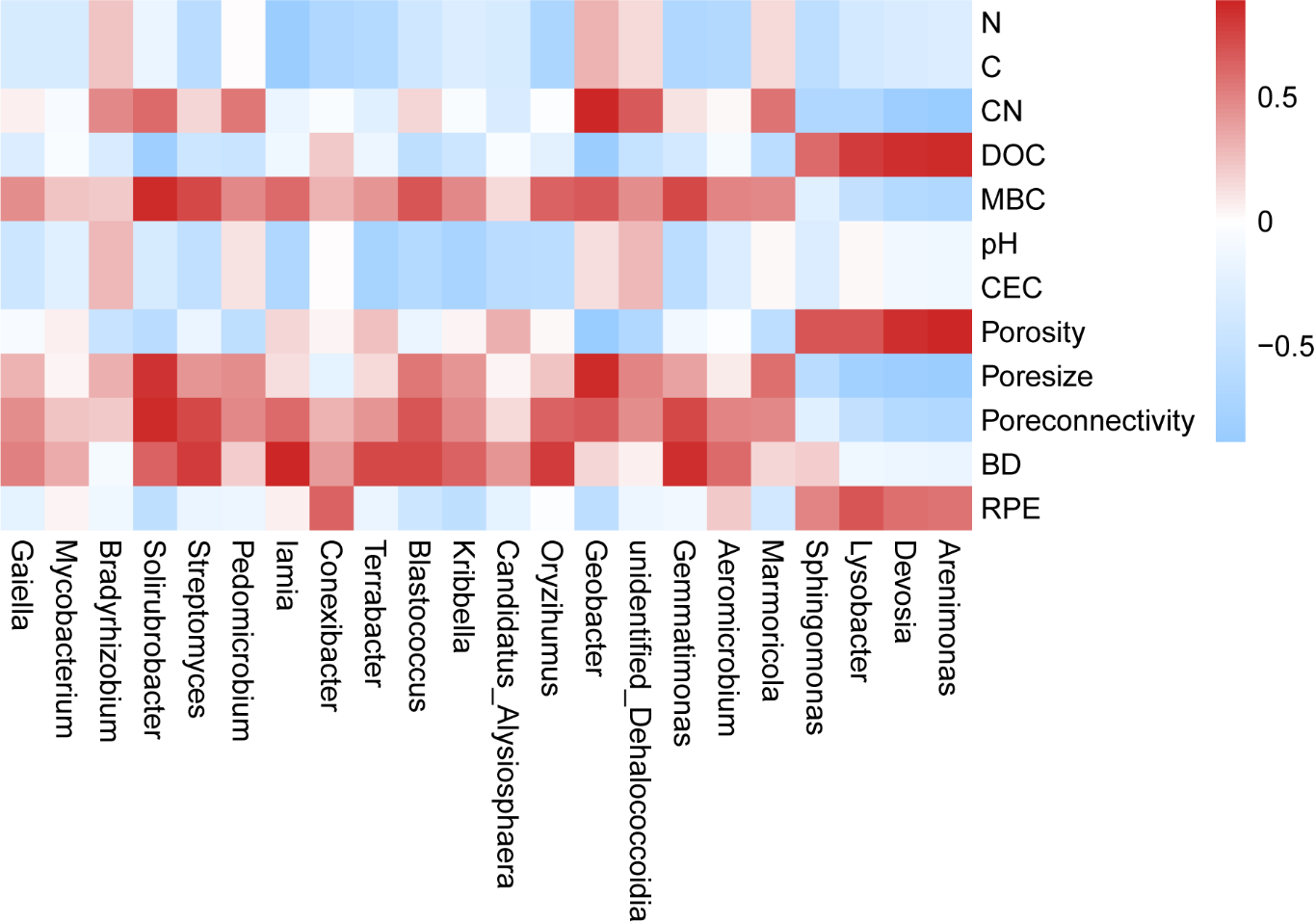

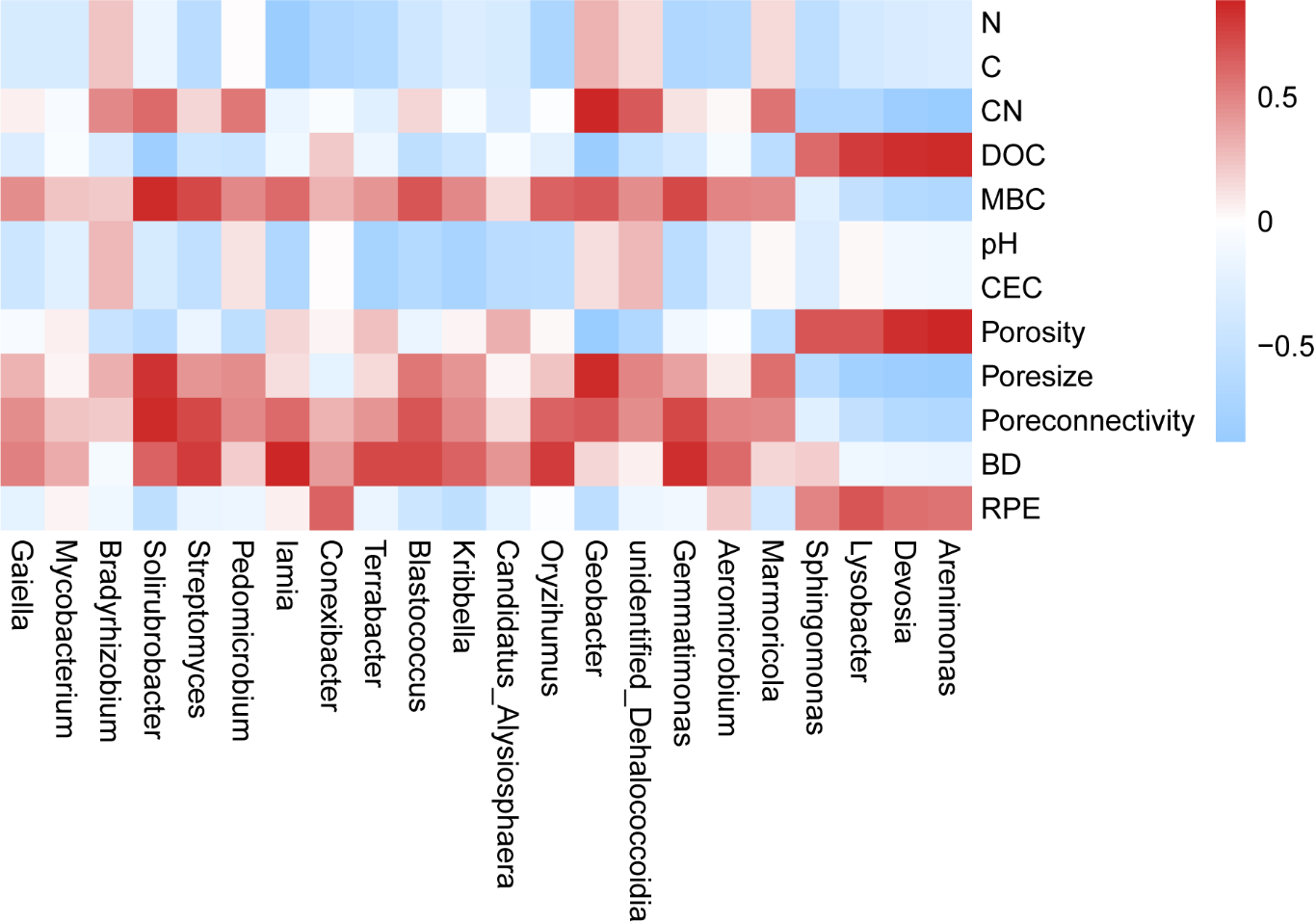


**Fig. S3.** Correlation coefficients between the most abundance genera, soil properties under biochar-amended treatments. Abbreviations: MBC; Microbial biomass carbon, BD; Bulk density, AG1;>2mm aggregates, AG2;0.25-2mm aggregates, AG3;<0.25mm aggregates, C/N; Carbon Nitrogen ratio, DOC; Dissolved organic carbon, C; Total carbon, N; Total N, CEC; Cation exchange capacity. Leave only which affect strong, >0.5

**Fig. S4**


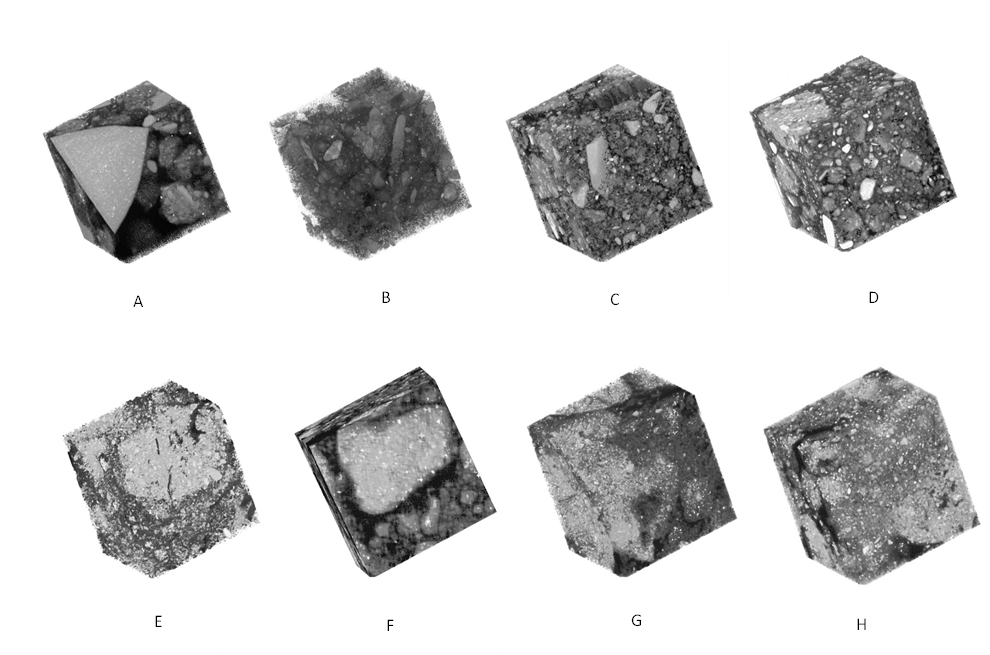

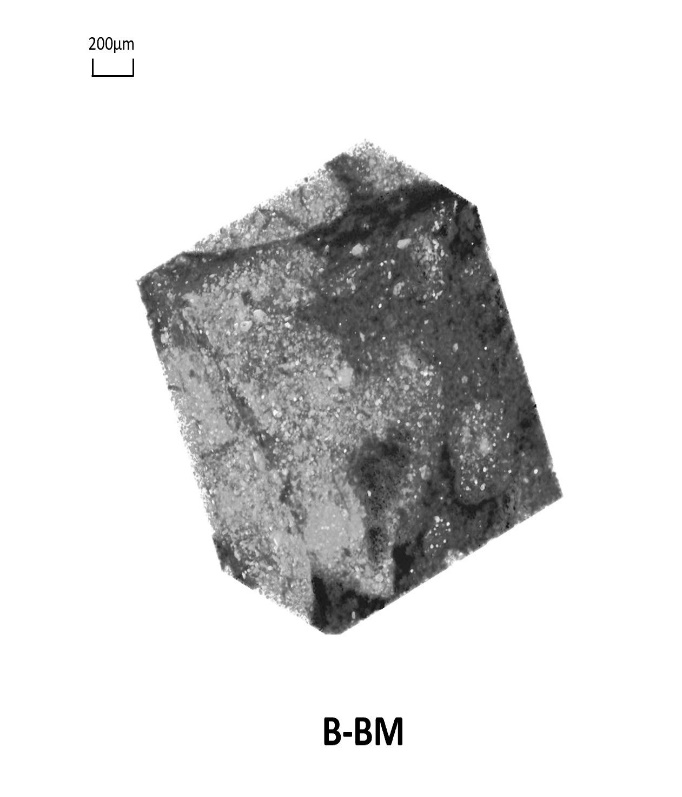


**Fig. S4.** Representative 2-D images of macroaggregates for the different Biochar treatments: (A) Control, (B) biochar low addition, (C) biochar medium addition, (D) biochar high addition for Cambisol soil. (E) Control, (F) biochar low addition, (G) biochar medium addition, (H) biochar high addition for Fluvisol soil. The quantification of pore characteristics was based on the selected square.

**Fig. S5**


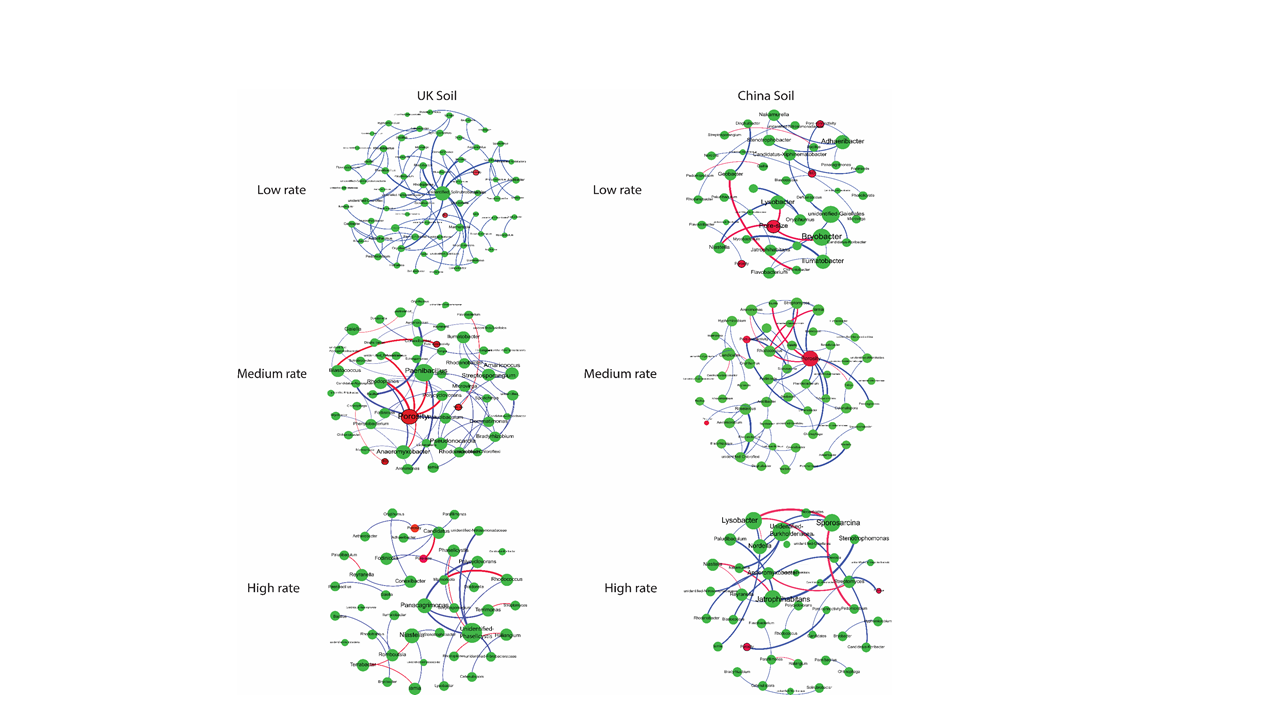

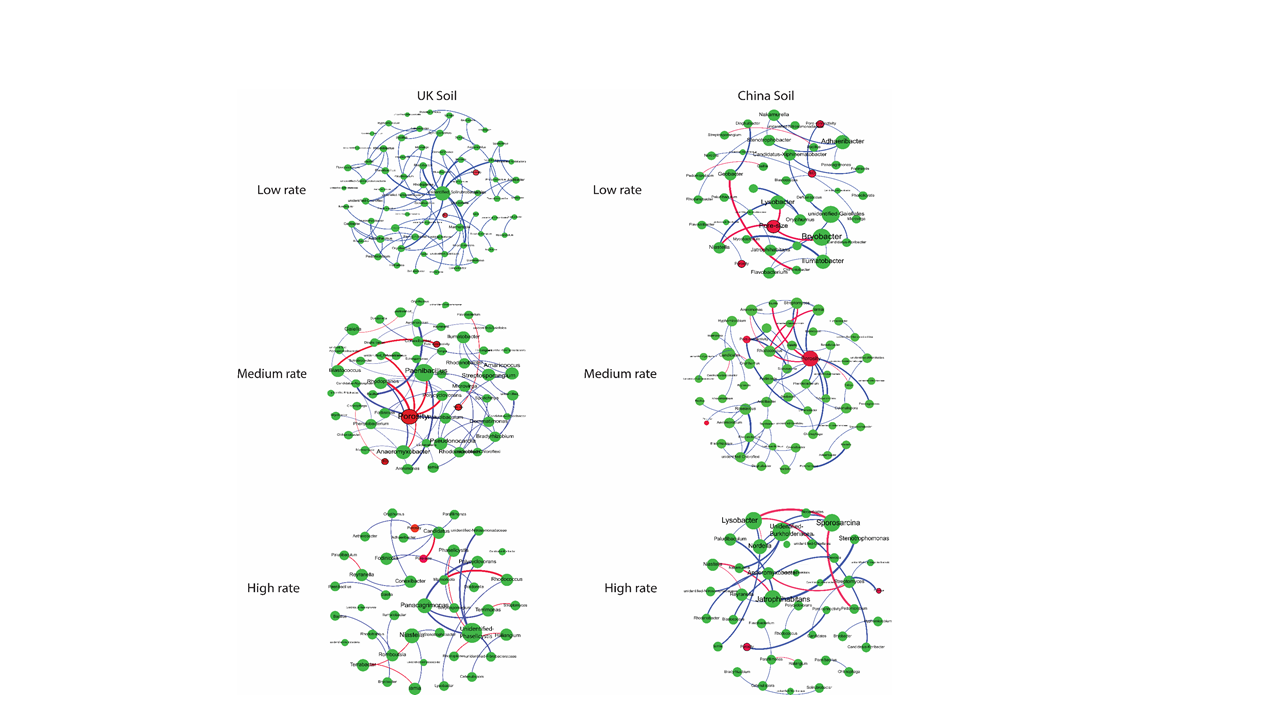


**Fig. S5.** Bacteria co-occurrence network and interaction with physiochemical properties (A,B) biochar low addition, (C,D) biochar high addition for Cambisol soil and Fluvisol soil. The quantification of pore characteristics was based on the selected square.
